# Increased renal elimination of endogenous and synthetic pyrimidine nucleosides in concentrative nucleoside transporter 1 deficient mice

**DOI:** 10.1038/s41467-023-38789-8

**Published:** 2023-06-01

**Authors:** Avinash K. Persaud, Matthew C. Bernier, Michael A. Massey, Shipra Agrawal, Tejinder Kaur, Debasis Nayak, Zhiliang Xie, Brenna Weadick, Ruchika Raj, Kasey Hill, Nicole Abbott, Arnav Joshi, Nadeen Anabtawi, Claire Bryant, Arpad Somogyi, Zobeida Cruz-Monserrate, Foued Amari, Vincenzo Coppola, Alex Sparreboom, Sharyn D. Baker, Jashvant D. Unadkat, Mitch A. Phelps, Rajgopal Govindarajan

**Affiliations:** 1 Division of Pharmaceutics & Pharmacology, College of Pharmacy, The Ohio State University, Columbus, OH 43210 USA; 2 Campus Chemical Instrument Center Mass Spectrometry and Proteomics Facility, The Ohio State University, Columbus, OH 43210 USA; 3 The Center for Life Sciences Education, College of Arts and Sciences, The Ohio State University, Columbus, OH 43210 USA; 4 Division of Nephrology & Hypertension, Renaissance School of Medicine, Stony Brook University, Stony Brook, NY 11794 USA; 5 Pharmacoanalytic Shared Resource (PhASR), The Ohio State University, Columbus, OH 43205 USA; 6 Center for Clinical & Translational Research, Nationwide Children’s Hospital, Columbus, OH 43210 USA; 7 Division of Gastroenterology, Hepatology, and Nutrition, College of Medicine, The Ohio State University, Columbus, OH 43210 USA; 8 Genetically Engineered Mouse Modeling Core, Ohio State University Comprehensive Cancer Center, The Ohio State University, Columbus, OH 43210 USA; 9 Department of Cancer Biology and Genetics, College of Medicine, The Ohio State University, Columbus, OH 43210 USA; 10 Department of Pharmaceutics, College of Pharmacy, University of Washington, Seattle, WA 98195 USA; 11 Translational Therapeutics, Ohio State University Comprehensive Cancer Center, Ohio State University, Columbus, OH 43210 USA

**Keywords:** Carrier proteins, Cancer therapeutic resistance, CRISPR-Cas9 genome editing

## Abstract

Concentrative nucleoside transporters (CNTs) are active nucleoside influx systems, but their in vivo roles are poorly defined. By generating CNT1 knockout (KO) mice, here we identify a role of CNT1 in the renal reabsorption of nucleosides. Deletion of CNT1 in mice increases the urinary excretion of endogenous pyrimidine nucleosides with compensatory alterations in purine nucleoside metabolism. In addition, CNT1 KO mice exhibits high urinary excretion of the nucleoside analog gemcitabine (dFdC), which results in poor tumor growth control in CNT1 KO mice harboring syngeneic pancreatic tumors. Interestingly, increasing the dFdC dose to attain an area under the concentration-time curve level equivalent to that achieved by wild-type (WT) mice rescues antitumor efficacy. The findings provide new insights into how CNT1 regulates reabsorption of endogenous and synthetic nucleosides in murine kidneys and suggest that the functional status of CNTs may account for the optimal action of pyrimidine nucleoside analog therapeutics in humans.

## Abstract (web summary)

Concentrative nucleoside transporters (CNTs) are cellular nucleoside influx systems, but their in vivo roles are poorly defined. By generating CNT1 knockout (KO) mice, here the authors show a role of CNT1 in the renal reabsorption of endogenous and synthetic nucleosides.

## Introduction

Nucleotides are essential for DNA replication, RNA synthesis, bioenergetics, and cellular signaling, and as a result, they are delicately regulated to maintain the optimal concentrations required to meet cellular demands^1^. Nucleotide biosynthesis and homeostasis are regulated by two processes: de novo nucleotide synthesis and salvage nucleotide synthesis. In de novo nucleotide synthesis, nucleotide bases are assembled from a series of simpler compounds. The nucleotide subclass of pyrimidines is assembled separately and then attached to ribose, whereas the nucleotide subclass of purines is synthesized in sections onto a ribose-based structure^2^. These elementary reactions are repeated with variation to generate each class of nucleotides. In salvage nucleotide synthesis, immediate nucleotide precursors, such as nucleobases and nucleosides, derived from DNA and RNA degradation and exogenous sources, are reclaimed before being converted back into nucleotides for biological use^2^. Among these two processes, the de novo pathway is better characterized because the underlying enzymatic reactions can be evaluated in cell-based assays and animal models; however, not all components within the salvage nucleotide pathway are well understood due to the lack of suitable models to evaluate their roles within the complex physiological system.

In mammals, the salvage nucleotide pathway is activated in many cell types, including those in skeletal muscle, bone marrow, brain, liver, intestine, and lymphoid tissues^3^. Earlier studies have identified several players involved in salvage nucleotide synthesis and, by extension, the maintenance of nucleotide homeostasis^4^. Nucleic acids are hydrolyzed into their nucleotides by nucleases, and nucleosidases and phosphatases further breakdown the resulting nucleotides to form nucleosides^5^. Nucleosides reenter the cell through nucleoside transporters^6^, and nucleoside kinases further convert nucleosides into nucleotides to regulate the cellular balance of the nucleotide pool. While the functions of the enzymatic components in the salvage nucleoside pathway have been well studied, mutations in these enzymes are known to cause severe human inborn errors of metabolism^7–10^. The roles played by solute carrier (SLC) transporters are often overlooked, and thus, their involvement in the dysregulation of nucleotide homeostasis that contributes to genomic instability, aberrant cell signaling, or metabolic disorders is not fully understood^11–15^. Among the two families of SLCs (i.e., equilibrative nucleoside transporters (ENTs) encoded by the SLC29A family and concentrative nucleoside transporters (CNTs) encoded by the SLC28A family), very little is known about CNT function in vivo although in vitro data on CNTs reveal a role in high affinity and active cellular nucleoside influx^16–20^.

Concentrative nucleoside transporter 1 (CNT1), encoded by *SLC28A1*, is the first member of the CNT family and prefers pyrimidine nucleosides^16,19^. Although more restrictive in expression than ENTs, CNT1 is known to be highly expressed in well-differentiated epithelial cells lining major organs, including the pancreas, intestines, and kidneys at the apical surface^21–23^. Cell-based assays and Xenopus oocyte assays have demonstrated that CNT1 is a Na^+^:nucleoside symporter (transport occurs at a 1:1 stoichiometry) with an apparent K_m_ in the range of 10–30 μmol/L for endogenous pyrimidine nucleosides^16,20^. Other studies have also demonstrated CNT1 to be a putative tumor suppressor with chemosensitizer properties, as it influences tumor cell proliferation and drives the accumulation of several chemotherapeutic cargos inside cancer cells, including fluoropyrimidines (e.g., gemcitabine) and hypomethylating nucleoside drugs (azacytidine, decitabine, zebularine)^23–25^. Recently, two independent studies showed that functional mutations in *SLC28A1* are associated with an inborn error of metabolism uridine-cytidinuria (URCTU) in which urinary excretion of the endogenous CNT1 substrates uridine and cytidine was high^26,27^. Moreover, single nucleotide polymorphisms (SNPs) in *SLC28A1* are associated with altered bioavailability of the nucleoside analog drugs used to treat allograft rejection and hepatic toxicity when these drugs are used to treat non-small cell lung cancer (NSCLC)^28,29^. Furthermore, loss of CNT1 expression has been identified in both solid tumors and leukemia, where nucleoside analogs are used as first-line treatment agents^30–32^. Despite the possibility that CNT1 plays prominent roles in both nucleoside salvage and nucleoside drug disposition, the clinical significance and translation of such findings is limited due to the lack of an understanding of the physiological and pharmacological roles of CNT1 in vivo.

In this study, by applying CRISPR/Cas9 genome editing to generate global CNT1 knockout (KO) mice (*Slc28a1*^*−/−*^), we detailed the first characterization of CNT1 KO mice for the in vivo handling of endogenous and synthetic nucleosides. Our findings uncovered an indispensable role for CNT1 in the renal salvage of pyrimidine nucleosides and that loss of CNT1 activity increases urinary letdown of both endogenous and synthetic pyrimidine nucleosides in mice. CNT1 deletion in mice was not detrimental to mouse fertility or survival due to putative compensatory mechanisms but rendered the frontline chemotherapeutic nucleoside drug gemcitabine (dFdC) less effective in a syngeneic mouse model of pancreatic cancer by increasing the urinary clearance of the drug, leading to accelerated mortality.

## Results

### Generation and characterization of CNT1-deficient mice

**Fig. 1:**
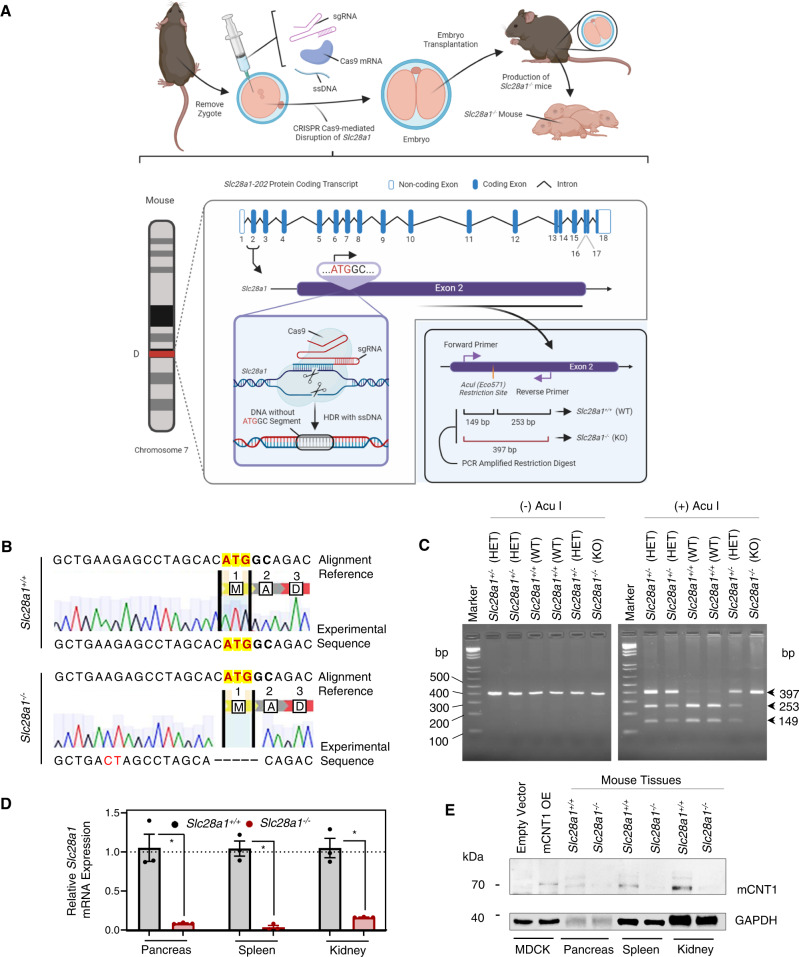
Generation and characterization of CNT1-deficient mice. **A** Schematic workflow illustrating the *Slc28a1* editing and genotyping strategy. **B** Representative DNA chromatogram illustrating the sequence alignment between the *Slc28a1* reference and experimental sequence from *Slc28a1*^+/+^ and *Slc28a1*^−/−^ mice with deleted nucleic acids indicated with a dash (-). Sequencing performed in two independent experiments. **C** Representative genotyping gel loaded with PCR-amplified *Slc28a1* from *Slc28a1*^+/+^ and *Slc28a1*^−/−^ with and without the Acul restriction digest. Genotyping performed in over ten independent experiments. **D** Relative *Slc28a1* gene expression in *Slc28a1*^−/−^ mouse organs normalized to *GAPDH* and *Slc28a1*^+/+^ control displayed as a fold change (2^−ΔΔCt^). Data represent mean ± SEM (*n* = 3 mice/group mean ± SEM, *p < 0.05 by two-tailed *t*-test). Gene expression performed in two independent experiments. **E** Representative immunoblots of mCNT1 in *Slc28a1*^+/+^ and *Slc28a1*^−/−^ mouse organs at 8 weeks of age with GAPDH as a loading control. Empty vector and mCNT1 overexpression (OE) controls were generated in HEK293 cells (#CRL 1573, ATCC). Immunoblotting performed in two independent experiments.

The mouse genome contains one *Slc28a1* gene, which is in the D3 region of chromosome 7 that encodes the CNT1 protein (ENSMUSG00000025726, Gene ID: 434203)^33,34^. Gene editing was conducted at the *Slc28a1* locus in C57BL/6NTac mouse embryos using CRISPR/Cas9 technology to target the ATG start codon located in exon 2 of *Slc28a1* (Fig. 1A). sgRNAs with a predicted “on-target” score > 50 and an “off-target” score ≥ 60 were selected to mutate the first translated exon (exon 2) of *Slc28a1*. The selected gRNA sequence provided minimal off-target effects, as evidenced by an off-target score of <1.73 **(**Supplementary Table 1**)**. A single strand (ss) DNA oligo lacking the start codon and two additional nucleotides (5′-ATGGC-3′) was used as a homology directed repair (HDR) template after CRISPR/Cas9-mediated cleavage of *Slc28a1*. In addition, a g.AG > CT mutation was introduced to disrupt the AcuI (Eco571) restriction site upstream of the start codon for genotyping purposes. Subsequent genomic DNA sequencing identified the successful deletion of the 5′-ATGGC-3′ DNA segment containing the *Slc28a1* start codon, which produced a frameshift mutation throughout the remainder of the *Slc28a1* transcript, and successful mutation of the AcuI restriction site (Fig. 1B). As a result, the enzymatic digest of PCR-amplified *Slc28a1* with the AcuI restriction enzyme produced two DNA products for C57BL/6NTac wild-type (WT) (*Slc28a1*^*+/+*^) mice at 149 bp and 253 bp, three DNA products for heterozygous (*Slc28a1*^*+/−*^) mice at 149 bp, 253 bp and 397 bp, and one DNA product for C57BL/6NTac CNT1 KO (*Slc28a1*^*−/−*^) mice at 397 bp, allowing for the identification of mice carrying WT or variant alleles (Fig. 1C). Evaluation of the *Slc28a1* transcripts in the mouse pancreas, spleen, and kidney identified a dramatic reduction in *Slc28a1*^*−/−*^ mice, likely due to nonsense-mediated mRNA decay (Fig. 1D). Western blotting analysis of lysates from these tissues showed a loss of mCNT1 protein expression (Fig. 1E). On higher exposure, however, a very faint band corresponding to the CNT1 migratory position was observed possibly due to a non-functional CNT1 protein transcribed from an RNA splice variant^35^. Nevertheless, densitometric quantification showed a >95% reduction in CNT1 immunoreactivity in the *Slc28a1*^*−/−*^ mouse tissues with no major alterations in the transcript levels of other nucleoside transporters (<2-fold change) (Fig. S1A).

We previously reported that mice deficient in an intracellular ENT3 (*Slc29a3*^−/−^) develop severe aplastic anemia, breaches of mesodermal tissue integrity and increased mortality at ~18–20 weeks^36^. Similarly, the loss of cell surface ENT1 (*Slc29a1*^−/−^) in mice was shown to alter bone density in older mice (~16–28 weeks) with aberrant mineralization in the cervicothoracic and lumbar spine and femur^37^. Unlike ENT3 or ENT1 KO mice, CNT1 KO (*Slc28a1*^*−/−*^) mice showed no obvious clinical abnormalities and a similar lifespan to *Slc28a1*^*+/+*^ (WT) mice after examination for ~100 weeks. *Slc28a1*^*−/−*^ mice also reproduced at a standard rate compared to *Slc28a1*^*+/+*^ mice with no apparent abnormalities in the *Slc28a1*^*−/−*^newborn mice. Comprehensive phenotyping of adult *Slc28a1*^*−/−*^ mice at approximately 20 weeks (at which stage the *Slc29a3*^−/−^ and *Slc29a1*^−/−^ mice demonstrated phenotypic abnormalities) showed no gross or histopathological lesions (compared to age-matched WT mice) in the kidney, intestine, pancreas, liver, spleen, or bone marrow, where CNT1 is normally expressed. The only histopathological abnormalities found were lymphoid aggregates in the salivary glands, follicular epithelial dysplasia of the thyroid gland, and hepatocellular glycogenosis in the liver, which are generally considered normal findings in C57BL/6NTac background strains.

Hematological analysis of *Slc28a1*^*−/−*^ mice at 20 weeks revealed significant decreases in the red blood cell (RBC) count and hematocrit levels with compensatory increases in the number of reticulocytes and the differential reticulocyte count (Fig. S1B); no significant changes were observed in white blood cells (WBCs) or platelets except for a modest non-significant decrease in neutrophils, eosinophils, and basophils (Fig. S1C). Anemia was less severe than that observed in ENT3 KO mice and did not cause any clinical abnormalities at any age or increase the mortality of *Slc28a1*^*−/−*^ mice. The serum biochemical profile at 20 weeks revealed a significant increase in serum creatinine (CREAT) in *Slc28a1*^*−/−*^ mice, which is indicative of renal impairment (Fig. S1D). However, blood urea nitrogen (BUN) and BUN:CREAT ratio did not change significantly, suggesting only mild renal impairment (Fig. S1D). Further, no albuminuria was observed (Fig. S1E), and the average urinary creatinine levels in *Slc28a1*^*−/−*^ mice were comparable to that of WT mice (Fig. S1F) without any substantial changes in albumin to creatinine ratio (Fig. S1G)^38^. The clinical chemistry of *Slc28a1*^*−/−*^ mouse serum revealed significant increases in calcium (CA) and cholesterol (CHOL) concentrations and a significant decrease in chloride (CL) without alterations to any proteins, enzymes, lipids, or other electrolytes (Fig. S1D).

Together, these results indicate the successful generation of a viable and fertile mouse model devoid of CNT1 that exhibits no overt clinical phenotypes except for mild anemia and renal impairment with some alterations to the blood biochemical parameters.

### High excretion of pyrimidine (deoxy)nucleosides in *Slc28a1*^*−/−*^ mouse urine

**Fig. 2:**
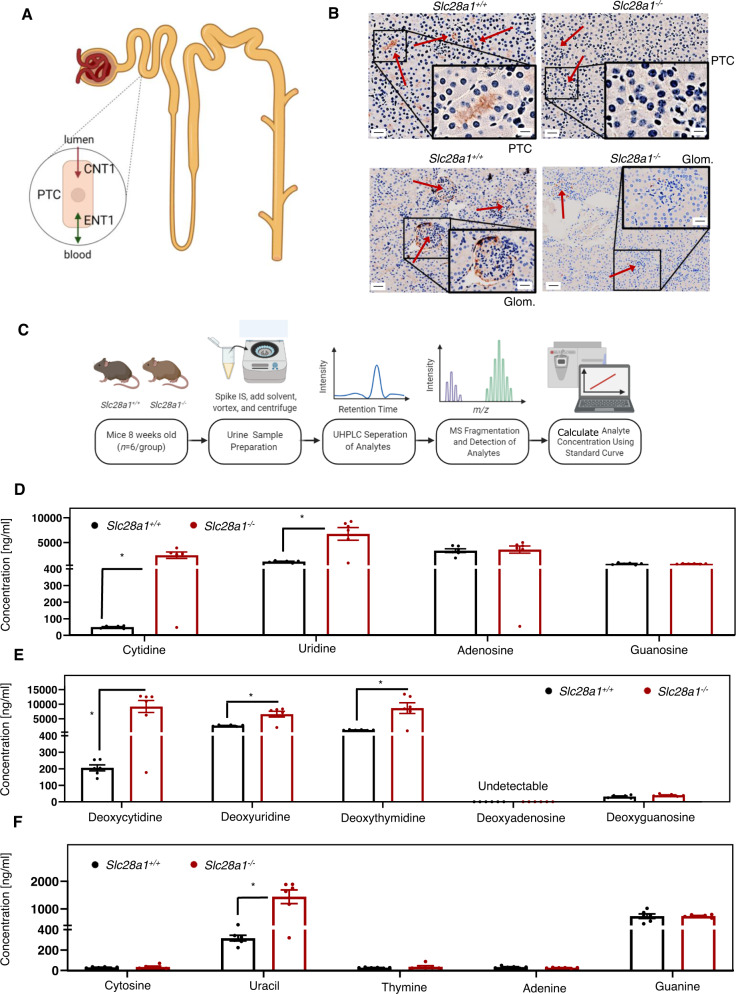
CNT1 kidney immunohistochemistry and targeted metabolomics of *Slc28a1*^*−/−*^ mice urine. **A** Diagram illustrating transporter localization in the kidney. PTC, proximal tubular cell; arrows indicate transport directionality of CNT1 and ENT1. **B** Representative CNT1 DAB-stained kidney section (×10; Scale bar = 100 μm) comparing *Slc28a1*^+/+^ with *Slc28a1*^−/−^ mice at 8 weeks. Insets shows higher magnification (×40; Scale bar = 20 μm) of boxed regions. CNT1 DAB staining is present at the apical surface of proximal tubule cells and glomerular cells (red arrows) in *Slc28a1*^+/+^ mice but not in the *Slc28a1*^−/−^ mice. Kidney imaging performed three independent experiments. **C** Schematic workflow used to profile the nucleobases, nucleosides, and (deoxy)nucleosides in *Slc28a1*^+/+^ and *Slc28a1*^−/−^ mice urine. **D** Concentration of nucleosides in *Slc28a1*^−/−^ mice urine compared to *Slc28a1*^+/+^ littermate control mice. Data represent mean ± SEM (*n* = 6 mice/group, *p < 0.05 by two-tailed *t*-test). **E** Concentration of (deoxy)nucleosides in *Slc28a1*^−/−^ mice urine compared to *Slc28a1*^+/+^ littermate control mice. Data represent mean ± SEM (*n* = 6 mice/group, *p < 0.05 by two-tailed *t*-test). **F** Concentration of nucleobases in *Slc28a1*^−/−^ mice urine compared to *Slc28a1*^+/+^ littermate control mice. Data represent mean ± SEM (*n* = 6 mice/group, *p < 0.05 by two-tailed t-test). Profiling of nucleobases, nucleosides, and (deoxy)nucleosides was performed in two independent experiments.

The apparent renal impairment identified in CNT1 KO mice was interesting and may be related to the increased urinary excretion of uridine and cytidine observed in humans with CNT1 mutations. We previously demonstrated that human CNT1 is localized at the apical surface of renal proximal tubular epithelial cells and the glomerular cells of human kidneys^39^. In addition, we and others have shown that CNT1 expression is localized to the apical surface of canine kidney epithelial cells and human embryonic kidney cells and, in cooperation with ENT1 localization at the basolateral surface, participates in the transepithelial flux of nucleosides across the renal epithelium^40,41^. Such a vectorial mode of transport could facilitate the reabsorption of nucleosides from urine formed within the proximal tubules (Fig. 2A), but in vivo evidence for such an occurrence is absent. When WT mouse renal sections were stained with 3,3′-diaminobenzidine (DAB) for immunohistochemical analysis of CNT1 protein expression, we observed CNT1 expression and localization along the apical surface of proximal tubule cells (Fig. 2B), like that observed in the human kidneys. In addition, some glomerular cells were positively stained for CNT1. The CNT1 expression was absent in *Slc28a1*^*−/−*^ mouse tubular and glomerular cells with only a faint background-like staining observed in a few glomeruli (Fig. 2B).

Like human CNT1, murine CNT1 transports both pyrimidine ribonucleosides and deoxyribonucleosides but not their purine counterparts^20,42^; thus, we investigated whether CNT1 loss in murine kidneys altered the renal reabsorption of nucleosides and therefore the urinary excretion of nucleosides. Using specific and targeted liquid chromatography–tandem mass spectrometry (LC–MS/MS) analysis, the concentrations of each nucleoside (adenosine, guanosine, uridine, cytidine) and deoxynucleoside (deoxyadenosine, deoxyguanosine, deoxyuridine, deoxycytidine, deoxythymidine) were measured in the urine of *Slc28a1*^*+/+*^ and *Slc28a1*^*−/−*^ mice. Because nucleoside phosphorylases and ribonucleotide reductases are abundant in the plasma and urine of mice^43^ and can convert (deoxy)nucleosides to their respective nucleobases, we also examined the levels of five nucleobases (adenine, guanine, uracil, cytosine, thymine) by targeted LC–MS/MS analysis. Furthermore, to reduce variability due to dietary origin and/or intestinal absorption of nucleosides, we starved the mice for 12 h before measuring these parameters.

Urine was collected during the 12 h starvation period from 8-week-old (age-matched) *Slc28a1*^*+/+*^ and *Slc28a1*^*−/−*^ mice (*n* = 6), immediately mixed with tetrahydrouridine (THU; 10 µg/ml) to prevent rapid deamination of the nucleosides and analyzed using time-of-flight mass spectrometry (TOFMS) with ultra-high performance liquid chromatography (UHPLC) (Fig. 2C). Linearity was established for analytes over the concentration ranges observed and the lower limit of quantification of the LC–MS/MS assay was approximately 0.1 ng/ml for each analyte (Fig. S2). Interestingly, the targeted analysis revealed increases in the contents of the nucleosides uridine, and cytidine by 5.9-, and 52.9-fold (Fig. 2D), respectively, and the (deoxy)nucleosides deoxyuridine, deoxycytidine, and deoxythymidine increased by 2.4-, 44.8-, and 7.5-fold (Fig. 2E), respectively, in the urine of *Slc28a1*^*−/−*^ mice collected over 12 h. Unlike pyrimidine (deoxy)nucleosides, the concentrations of the purine (deoxy)nucleosides (adenosine, guanosine) were not significantly different between the *Slc28a1*^*+/+*^ and *Slc28a1*^*−/−*^ mouse urine samples, which is consistent with the specificity of CNT1 to transport only pyrimidine nucleosides. Both deoxyadenosine and deoxyguanosine were undetectable in *Slc28a1*^*+/+*^ and *Slc28a1*^*−/−*^ deleted mice. No significant alterations in the urinary excretion of pyrimidine nucleobases were observed except for uracil, which was increased by 4.6-fold in *Slc28a1*^*−/−*^ deleted mice (Fig. 2F). Thus, the increased urinary excretion of several pyrimidine nucleosides in *Slc28a1*^*−/−*^ mice provides the direct support for CNT1’s role in the reabsorption of endogenous pyrimidine (deoxy)nucleosides in murine kidneys.

### Loss of CNT1 in mice alters the urine and plasma metabolomes

**Fig. 3:**
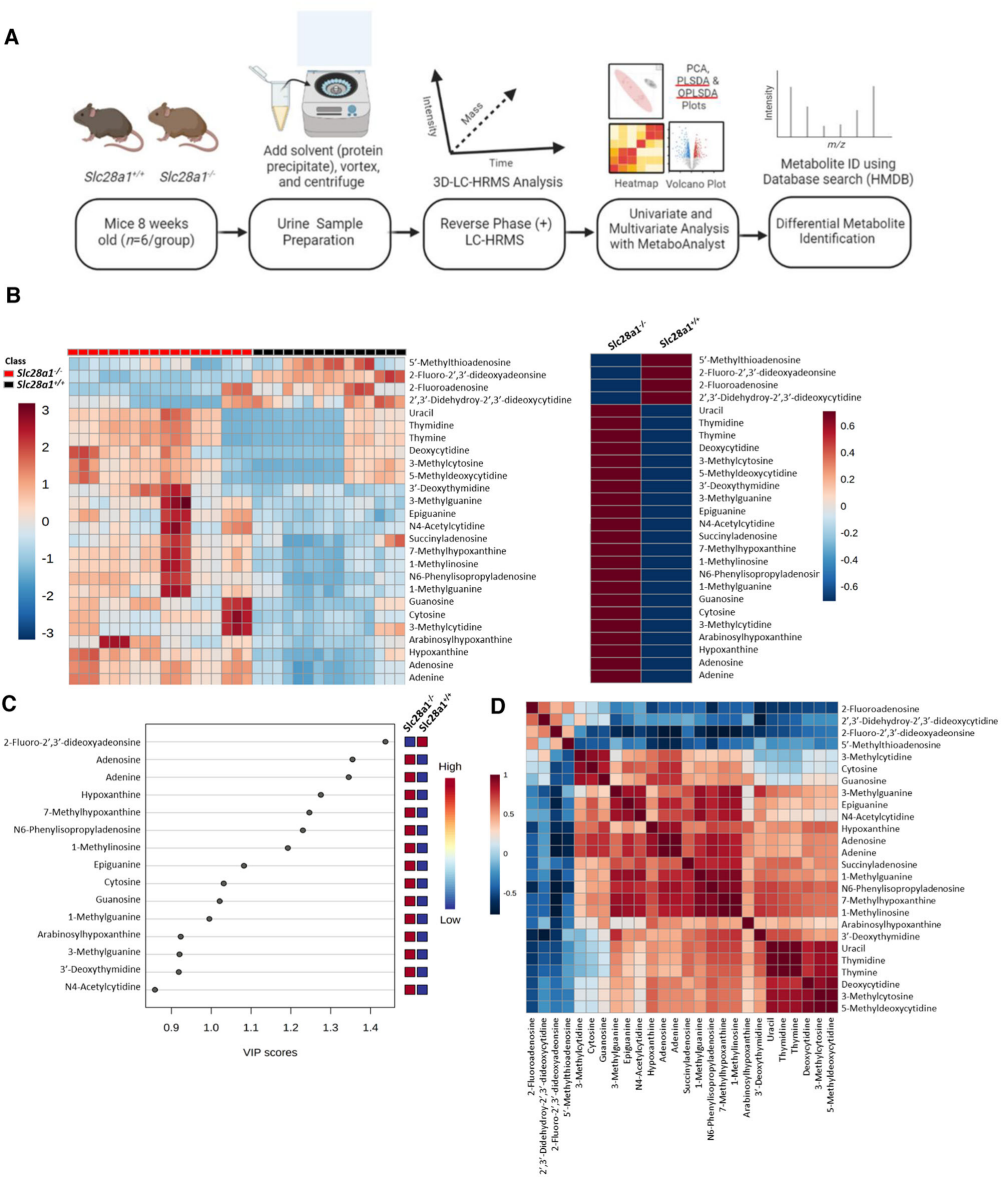
Untargeted metabolomics analysis of *Slc28a1*^−/−^ mice urine. **A** Schematic workflow used to profile the urine metabolome of *Slc28a1*^+/+^ and *Slc28a1*^−/−^ mice. **B** Heatmap illustrating hierarchical clustering of differential features (left) and the average abundances (right) for nucleoside derived metabolites detected across 5 *Slc28a1*^+/+^ and 6 *Slc28a1*^−/−^ mice urine samples run in triplicate by mass spectrometry-based metabolomics. Data represent mean ng/ml ± SEM (*n* = 5–6 mice/group mean ± SEM, *p < 0.05 by two-tailed *t*-test). MS signal intensities for all heatmaps were clustered in two dimensions based on Euclidean distance (*row*, metabolites; *column*, samples). Colors indicate the metabolite abundances (red, high; blue, low). For identified metabolites, increased (*red*) or decreased (*blue*) fold change in *Slc28a1*^−/−^ and corresponding *p* value (black) indicated. **C** VIP (Variable Importance in Projection) Scores for annotated nucleoside derived features in partial Least Squares-discriminant Analysis (PLS-DA). **D** Correlation heatmap illustrating the overall correlation between different features. Untargeted metabolomics was performed in one experiment with 5–6 mice per experimental group.

To understand the global consequences of CNT1 loss especially in the normal physiological state in mice (i.e., without starvation), we next conducted MS-based unbiased metabolomics analysis on *Slc28a1*^*−/−*^ mouse urine to detect and profile small molecules at ad libitum (Fig. 3A). We detected 4,352 total features in the urine of *Slc28a1*^*−/−*^ mice, among which 2,740 metabolites were significantly altered compared to their contents in the urine of *Slc28a1*^*+/+*^ mice, both collected over 24 h (*p* ≤ 0.05 and fold change ≥ 2.0; *n* = 6). Univariate and multivariate analyses of the untargeted metabolomics data revealed widespread alterations in the urinary levels of nucleobases, nucleosides, and (deoxy)nucleosides (Fig. 3B and S3; Supplementary Data S1). Strikingly, 22 out of 26 total detected nucleoside features showed an increase in the 0-24 h urine sample of *Slc28a1*^*−/−*^ mice. Like targeted analysis, unbiased metabolomics analysis also showed elevated urinary excretion of deoxycytidine and deoxythymidine (deoxycytidine-2.67-fold, deoxythymidine-2.05-fold) but in addition, increases in their derivatives (5’-methyldeoxycytidine-2.04-fold, 3′-methylcytosine-2-fold, thymine-2.05-fold) were also observed in untargeted analysis. Interestingly, cytidine levels showed no change, which suggests that cytidine is either efficiently reabsorbed in the WT kidneys by CNT1 during starvation (therefore higher levels were observed in the KO urine during starvation) or not significantly eliminated through murine kidneys at ad libitum. However, the increase in the level of the corresponding nucleobase cytosine along with the increase in 3-methylcytosine in the urine of *Slc28a1*^*−/−*^ mice at ad libitum also suggests that cytidine is at least partially metabolized to cytosine with subsequent conversion to 3-methylcytosine^44,45^. Likewise, there were no changes in the uridine contents, but the corresponding nucleobase uracil increased in the urine of *Slc28a1*^*−/−*^ mice, which was however like that observed at starvation.

Interestingly, increases in some of the purine metabolites (arabinosylhypoxanthine-2.4-fold, adenine-2.03-fold, adenosine-2.04-fold, N6-phenylisopropyladenosine-2.32 fold) were observed in *Slc28a1*^*−/−*^ mouse urine at ad libitum. Furthermore, in addition to nucleosides and their associated metabolites, high levels of cortisol and their derivatives (18-oxocortisol, hydroxycortisol, tetrahydrocortisol, 11-deoxycortisol,) as well as a few prostaglandins (F2α and D1) were prominent in *Slc28a1*^*−/−*^ mouse urine (Supplementary Table 2; Supplementary Data S1). Creatine or creatinine levels remained unaltered, but, amino acids (L-proline), dipeptides (glu-ile, glu-met, trp-asn), amino acid conjugates (acetylcysteine, N-acetyl phenylalanine, thioproline) and several carnitine derivatives were among the top altered metabolites. Taken together, the urinary metabolome analysis in *Slc28a1*^*−/−*^ mice at ad libitum identified that the increased elimination of pyrimidine nucleosides was accompanied by increased conversion of (deoxy)nucleosides to their corresponding (deoxy)nucleobases or conjugated metabolites with increased excretion of purine nucleoside, amino acid, carnitine, and cortisol derivatives **(**Supplementary Table 2; Supplementary Data S1).

To understand the systemic handling of nucleosides by CNT1, we simultaneously evaluated metabolite features in *Slc28a1*^*+/+*^ and *Slc28a1*^*−/−*^ mouse plasma using the same mice and conditions as those for the unbiased urinalysis (Fig. S5). Plasma was collected from mice by blood sampling at the end of the 24 h urine collection period at ad libitum. Untargeted metabolomics data analysis detected 2051 total features in the plasma of *Slc28a1*^*−/−*^ mice, among which 395 metabolites were significantly altered when compared to the plasma of *Slc28a1*^*+/+*^ mice (*p* ≤ 0.05 and fold change ≥ 2.0). Comparatively, the overall magnitude changes in the metabolite features in the *Slc28a1*^−/−^ mouse plasma were less pronounced (<5-fold changes) suggesting the impact of CNT1 loss is maximal in the urine at ad libitum. Univariate and multivariate analyses revealed alterations in the plasma levels of certain nucleosides, amino acids, fatty acids, carnitines, and their derivatives in *Slc28a1*^*−/−*^ mouse plasma **(**Fig. S5; Supplementary Data S2). Despite CNT1 does not transport purine (deoxy)nucleosides, the levels of adenosine, adenosine monophosphate, hypoxanthine, arabinosylhypoxanthine were decreased in *Slc28a1*^*−/−*^ mouse plasma which also corresponded to the increases in the same metabolites in *Slc28a1*^*−/−*^ mouse urine at ad libitum. As opposed to the increases in carnitine derivatives in the *Slc28a1*^−/−^ urine, decreases in the levels of carnitine derivatives (i.e., hexanoylcarnitine, propionylcarnitine) are observed in *Slc28a1*^−/−^ plasma. Besides some long-chain fatty acids, a few amino acids (tyr, trp) and small peptides (met-lys-lys, asn-gly-lys-gly), as well as corticosterone showed modest increases in *Slc28a1*^−/−^ mouse plasma. Thus, the plasma metabolome analyses of CNT1-deficient mice at ad libitum indicated decreases in purine nucleosides and carnitine derivatives along with the increases in certain amino acids, peptides, fatty acids, and corticosterone (Supplementary Table 3**;** Supplementary Data S2).

**Fig. 4:**
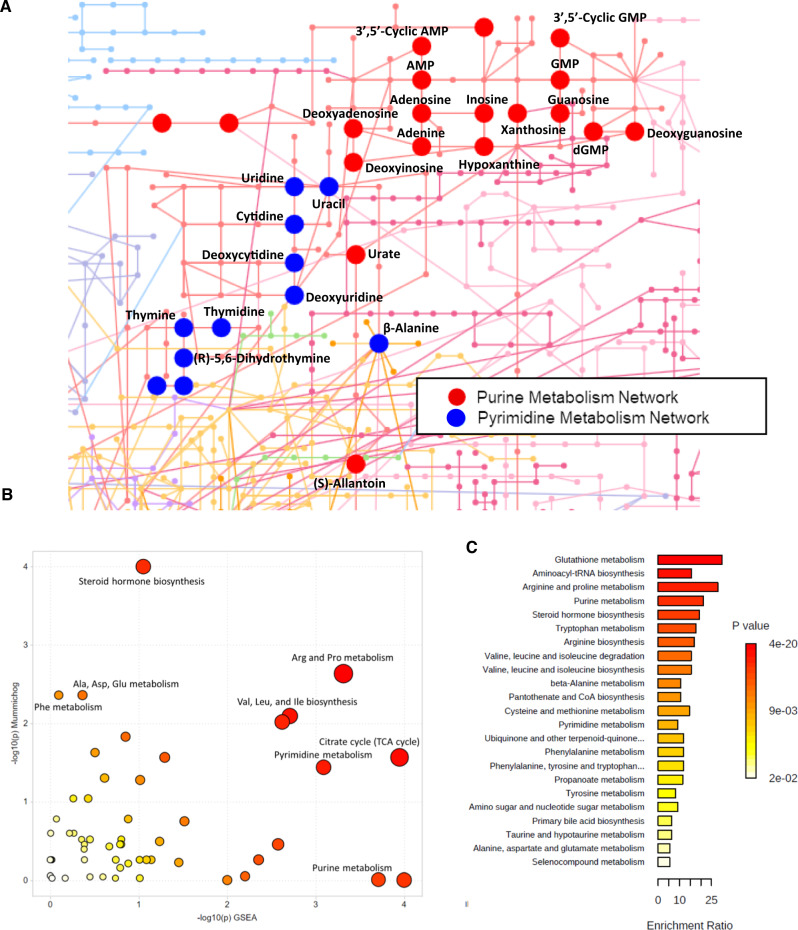
Untargeted metabolite pathway analysis of *Slc28a1*^*−/−*^ mice urine. **A** Network visualization of the purine and pyrimidine metabolite networks with altered purine metabolites highlighted red and altered pyrimidine metabolites highlighted in blue for urine metabolomics data using Fisher’s method of MS Peaks-to-Paths analysis, **B** the Mummichog and GSEA pathway Meta-analysis for MS Peaks to Paths combining the separate algorithms’ *p* values (*p < 0.05 by one-tailed hypergeometric test), and **C** Quantitative enrichment analysis using the concentration table of the final annotated list of features for the untargeted differential analysis for *Slc28a1*^−/−^ vs *Slc28a1*^−/−^ mice urine using the HMDB codes for each feature and the KEGG library (*p < 0.05 by two-tailed Welch’s *t*-test).

To collaboratively evaluate the metabolomics data derived from both plasma and urine, the *mummichog* algorithm and an adapted gene set enrichment analysis (GSEA) method were employed to identify global pathway activity changes in *Slc28a1*^*−/−*^ mice at ad libitum^46,47^. Metabolomics data, including the *m/z* features, *p* values, and statistical scores, from the *Slc28a1*^*+/+*^ and *Slc28a1*^*−/−*^ mouse urine and plasma samples were integrated into the MS Peaks to Pathways module of MetaboAnalyst 4.0, and the *mummichog* algorithm was then applied to generate mummichog pathway analysis plots with a global KEGG metabolic network. The mummichog pathway analysis plots display all the matched pathways as circles, with the color and size of each circle corresponding to its *p* value and enrichment factor, respectively, where the enrichment factor is the ratio between the number of significant (*p* < 0.001) pathway hits and the expected number of pathway hits. The urine pathway analysis revealed 53 matched pathways, with pyrimidine and purine metabolism being among the top hits (Fig. 4A). A total of 11 differential metabolites were produced during pyrimidine metabolism and were present in the urine of *Slc28a1*^*−/−*^ mice compared to *Slc28a1*^*+/+*^ mice, including cytidine, thymidine, uridine, and β-ala. Moreover, 19 differential metabolites produced during purine metabolism were detectable in the urine of *Slc28a1*^*−/−*^ mice, including adenosine, xanthine, guanosine, allantoin, and urea (Fig. 4A). The plasma pathway analysis revealed 17 matched pathways, with purine metabolism pathways being among the top hits (Fig. S5). There were a total of 12 differential metabolites produced during purine metabolism present in the plasma of *Slc28a1*^*−/−*^ mice when compared to *Slc28a1*^*+/+*^ mice, including adenosine and purine metabolic byproducts, xanthine and urate **(**Fig. S5**)**. In both the urine and plasma pathway analyses, purine metabolism was identified as one of the top altered pathways in *Slc28a1*^*−/−*^ mice (Figs. 3, 4, and S5). The mummichog analysis containing all ranked pathways enriched in *Slc28a1*^*−/−*^ urine and plasma is tabularized in the Supplementary materials (Supplementary Tables 4 and 5).

### ***Slc28a1***^***−/−***^**mice exhibit decreased plasma exposure to nucleoside drug due to urinary loss**

**Fig. 5:**
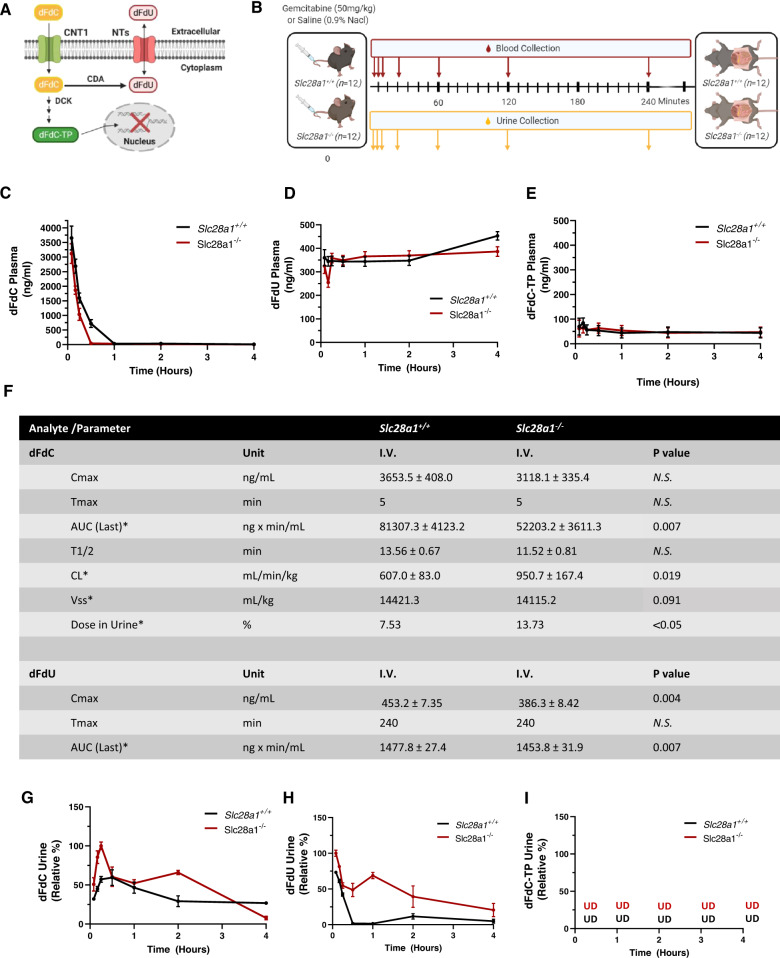
Plasma and urine pharmacokinetic profiling of dFdC and its metabolites. **A** Schematic diagram illustrating the mechanisms of action and metabolism of dFdC. **B** Schematic workflow illustrating *Slc28a1*^+/+^ and *Slc28a1*^−/−^ mouse treatment and serial blood and urine collection after dFdC administration. Plasma pharmacokinetic profiling of **C** dFdC, **D** dFdU, and **E** dFdC-TP in *Slc28a1*^+/+^ (*black*) and *Slc28a1*^−/−^ (*red*) mice receiving a single intravenous dose of dFdC (50 mg/kg). **F** Concentration-time data were analyzed by noncompartmental analysis, and pharmacokinetic parameters were calculated with WinNonlin (**F**, Table). Table shows plasma and urine pharmacokinetic parameters of dFdC and dFdU in *Slc28a1*^+/+^ and *Slc28a1*^−/−^ mouse plasma. Abbreviations: *C*_max_ maximum plasma concentration, *T*_max_, time at which the maximum plasma concentration is achieved, AUC area under the plasma concentration-time curve, *T*_1/2_ the half-life of the terminal phase, CL systemic clearance, Vss volume of distribution at steady state, NS not significant. Urinary pharmacokinetic profiling of **G** dFdC, **H** dFdU, and **I** dFdC-TP in *Slc28a1*^+/+^ (black) and *Slc28a1*^−/−^ (red) mice receiving a single intravenous dose of dFdC (50 mg/kg). Serial blood and urine sampling was performed at 5, 10, 15, 30, 60, 120, and 240 min, and analyte plasma and urine concentrations were determined by LC–MS/MS. Data represent the mean ± SEM (*n* = 6 mice/group). Plasma and urine pharmacokinetic profiling was performed in two independent experiments. *Footnote: (*p < 0.05 by two-tailed Welch’s *t*-test).

The lack of CNT1 leads to increased urinary excretion of endogenous nucleosides in *Slc28a1*^*−/−*^ mice; therefore, we next asked whether the loss of CNT1 would decrease systemic concentrations of nucleoside drugs, leading to reduced therapeutic efficacy. To test this hypothesis, we examined the plasma and urinary pharmacokinetic profiles of gemcitabine (dFdC), a fluoro substituted 2′-deoxycytidine chemotherapeutic with high affinity for CNT1 (*K*_m_ ∼12–36 μmol/L) that is currently used to treat pancreatic cancer and several other solid tumors (43–47). dFdC was also chosen as a probe substrate for CNT1 because of its limited diffusional uptake (Log *P* = −1.5) and limited transport by nonnucleoside transporters^48–52^. Intracellularly, dFdC is phosphorylated to become its primary active metabolite dFdC triphosphate (dFdC-TP) or deaminated to become its predominantly inactive metabolite 2′,2′-difluorodeoxyuridine (dFdU), which is readily excreted from the body (Fig. 5A)^53^. In addition to dFdC, we earlier reported dFdU is also a substrate for CNT1 and contributes to the anticancer activity of dFdC to a lesser degree^54^. As dFdC has been used at a dose of between 25–75 mg/kg in numerous preclinical mouse studies, the mean plasma concentration-time profiles for dFdC, dFdU, and dFdC-TP were quantified after a single intravenous injection of 50 mg/kg dFdC to determine whether CNT1 loss contributes to reduced systemic exposure in *Slc28a1*^*−/−*^ mice **(**Fig. 5B). Female *Slc28a1*^*+/+*^ and *Slc28a1*^−/−^ mice (8–12 weeks old) were intravenously dosed with dFdC, and blood samples were collected at seven time points (5, 10, 15, 30, 60, 120, and 240 min) into heparinized capillary tubes, mixed with THU, and centrifuged (1500 *× g* for 5 min). The resulting plasma supernatant was analyzed for dFdC, dFdU, and dFdC-TP using UHPLC–MS/MS as described in “Methods” (Figs. S6 and S7**)**.

Following intravenous administration, dFdC reached initial concentrations (*C*_max,_ peak plasma concentration) of 3635 ng/ml and 3118 ng/ml in *Slc28a1*^*+/+*^ and *Slc28a1*^*−/−*^ mouse plasma, respectively, at ~5 min in both groups (Fig. 5C). dFdC was rapidly converted to its primary deaminated metabolite, dFdU. A peak concentration for dFdU was indeterminable because of the long half-life of dFdU (~26 h)^53^ and the short duration of our analysis period (Fig. 5D). Additionally, we did not detect appreciable quantities of the primary active metabolite of dFdC, dFdC-TP, which is largely retained intracellularly and not readily detected in the plasma (Fig. 5E). dFdC plasma concentrations declined mono-exponentially with a terminal half-life of approximately 13.6 min in *Slc28a1*^*+/+*^ mice (Fig. 5F), which is similar to what was reported in a similar strain of strain of mice (B6C3F1) for dFdC (16.8 min)^55^. Interestingly, the terminal half-life (*T*_1/2_) of dFdC was non-significantly reduced in *Slc28a1*^*−/−*^ mice (11.2 min) compared to their age- and sex-matched *Slc28a1*^*+/+*^ counterparts (Fig. 5F). The reduced terminal half-life corresponded to significantly decreased systemic exposure to dFdC (represented as area under the plasma concentration-time curve (AUC) values) in *Slc28a1*^*+/+*^ and *Slc28a1*^*−/−*^ mice, which were 81307 ng × min/ml and 52203 ng × min/ml, respectively (Fig. 5F). This decrease in the AUC is associated with the significant increase in clearance observed in *Slc28a1*^*−/−*^ mice, which was 950 ml/min/kg compared to 607 ml/min/kg in *Slc28a1*^*+/+*^ mice (Fig. 5F). The noncompartmental pharmacokinetic parameters for dFdC in plasma are summarized in Fig. 5F.

Since dFdC is cleared faster in *Slc28a1*^*−/−*^ mice, it is possible that the increased urinary excretion of dFdC contributed to this loss analogous to increased excretion of endogenous nucleosides. We initially examined the pooled urine samples collected between 10 min and 2 h after dFdC administration from the same mice used for the plasma pharmacokinetic study. The mean concentrations of dFdC and dFdU in *Slc28a1*^*−/−*^ mouse urine appeared higher than the mean concentrations of dFdC and dFdU compared to their age- and sex-matched *Slc28a1*^*+/+*^ counterparts receiving an identical dose of dFdC whereas the levels of dFdC-TP were below the limits of quantification in both WT and KO mouse urines. Based on these trends, we speculated that urinary excretion of dFdC and dFdU is the primary mechanism contributing to the reduced dFdC plasma exposure in *Slc28a1*^*−/−*^ mice. To corroborate this further, the renal excretion of dFdC and dFdU was determined after a single intravenous injection of 50 mg/kg dFdC in an independent batch of mice (*n* = 6). Quantitation of dFdC and dFdU in urine at different time points (5, 10, 15, 30, 60, 120, and 240 min) was accomplished by preparing a 50-fold, or higher, dilution of urine samples in blank plasma and analyzing those diluted samples using UHPLC–MS/MS as described in Methods. Analyses of urinary levels revealed increased renal excretion of dFdC in the 10 min to 48 h collected urine of *Slc28a1*^*−/−*^ mice (13.74 % of total dose administered) compared to their age- and sex-matched *Slc28a1*^*+/+*^ counterparts (7.53 % of total dose administered) receiving an identical dose of dFdC (Fig. 5G–I). The non-compartmentally calculated renal clearance (CL_r_) of dFdC in *Slc28a1*^*−/−*^ mice was significantly higher (126.63%) than that of the renal clearance in *Slc28a1*^*+/+*^ mice, respectively. Taken together, these findings demonstrated that the loss of CNT1 reduced dFdC plasma exposure and increased its urinary excretion (along with its primary metabolite dFdU **(**Fig. 5H)), which further corroborated that urinary excretion is the primary mechanism contributing to the reduced dFdC plasma exposure in *Slc28a1*^*−/−*^ mice.

### Anticancer efficacy is compromised in *Slc28a1*^*−/−*^ mice, but chemotherapeutic dose optimization rescued these defects

**Fig. 6:**
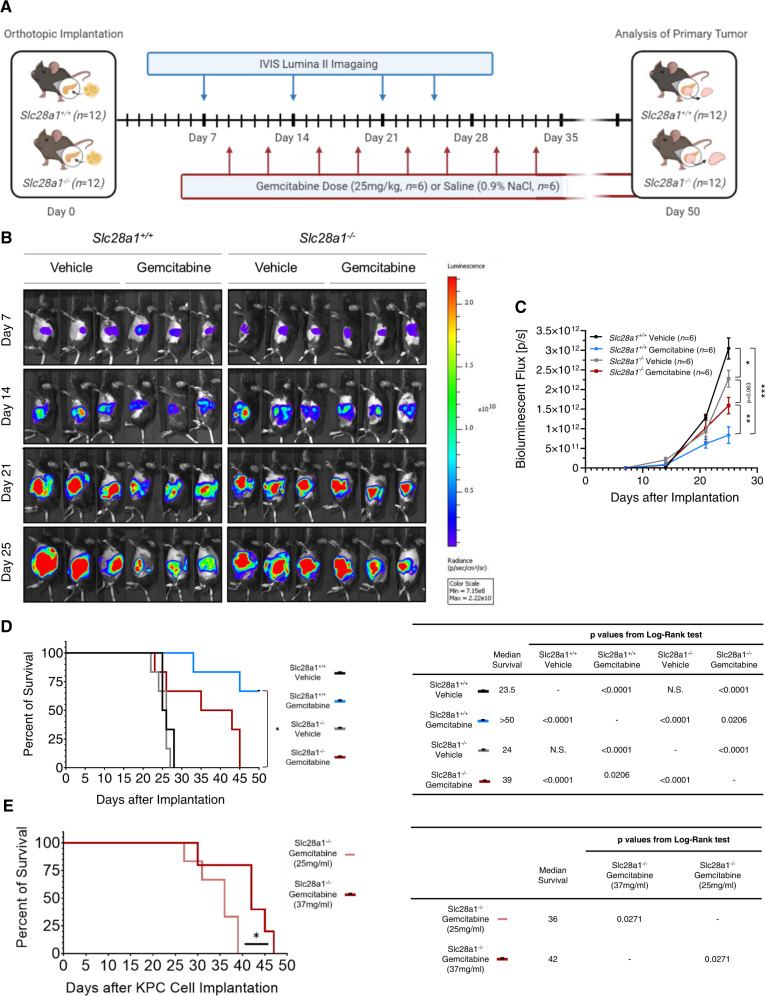
Gemcitabine antitumor efficacy in *Slc28a1*^*−/−*^ mice orthotopically implanted with pancreatic ductal adenocarcinoma cells. **A** Schematic workflow illustrating the *Slc28a1*^+/+^ and *Slc28a1*^−/−^ mouse treatment and imaging regimens post implantation. **B** Bioluminescent imaging of the total tumor burden in 12 *Slc28a1*^+/+^ and 12 *Slc28a1*^−/−^ mice 7, 14, 21, and 25 days post implantation with and without dFdC treatment (25 mg/kg, i.v.; 6 mice/group within each genotype; data shown for 3 representative mice/group). **C** Total bioluminescent flux [p/s] data collected over time from 6 *Slc28a1*^+/+^ and 6 *Slc28a1*^−/−^mice treated with and without dFdC (25 mg/kg, i.v.). Data represent the mean ± SEM (*n* = 6 mice/group, *p < 0.05 by two-tailed *t*-test). **D** Kaplan–Meier analysis of the 50-day survival after the orthotopic transplantation of pancreatic ductal adenocarcinoma cells into 6 *Slc28a1*^+/+^ and 6 *Slc28a1*^−/−^ mice, which were then treated twice a week with dFdC (25 mg/kg, i.v.). Statistical comparisons were completed using the Mantel-Cox test (*n* = 6/group, ***p < 0.001, Mantel–Cox test). **E** Kaplan–Meier analysis of 50-day survival after transplanting orthotopically implanting pancreatic ductal adenocarcinoma cells in 6 *Slc28a1*^−/−^ mice treated twice a week with 25 mg/kg, IV and 6 *Slc28a1*^−/−^ mice treated twice a week with 37 mg/kg, IV. Statistical comparisons were calculated using the Mantel–Cox test (*n* = 6/group; ***p < 0.001; Mantel–Cox test).

Given the essential role that CNT1 plays in retaining dFdC in systemic circulation and biological tissues and the need to further evaluate the contribution of CNT1 to dFdC therapeutic efficacy, we next examined whether the loss of CNT1 alters dFdC drug efficacy in a syngeneic mouse model of pancreatic cancer. Notably, orthotopic injection of a Kras/p53-mutated mouse PDAC cell line (KPC) into syngeneic WT mice allows the rapid development of pancreatic tumors with a severe metastatic burden that results in complete mortality within 3–5 weeks of implantation^56^, and administration of dFdC dramatically reduces tumor burden to delay mortality. Notably, the KPC cells express multiple nucleoside transporters capable of transporting dFdC (e.g., ENT1, ENT2). Therefore, 1 × 10^6^ KPC cells expressing luciferase were orthotopically implanted into the pancreases of *Slc28a1*^*+/+*^ and *Slc28a1*^*−/−*^ mice using the surgical procedure described in the Methods. All mice developed detectable pancreatic tumors 1 week post implantation and were subsequently dosed with dFdC (25 mg/kg, i.v.) or vehicle (saline, 0.9% NaCl) and imaged with an IVIS Lumina II system following a regimented schedule **(**Fig. 6A). Both *Slc28a1*^*+/+*^ and *Slc28a1*^*−/−*^ vehicle control mice developed large pancreatic tumors at 25 days post implantation. There was a minor, but significant, decrease in the total tumor burden in *Slc28a1*^*−/−*^ mice receiving vehicle compared to the control receiving vehicle, suggesting that CNT1 KO environment affected tumor burden in a tumor cell nonautonomous manner (Fig. 6B, C). While both *Slc28a1*^*+/+*^ and *Slc28a1*^*−/−*^ dFdC-treated mice exhibited reduced tumor growth over time, only *Slc28a1*^*+/+*^ mice and not *Slc28a1*^*−/−*^mice responded effectively to dFdC treatment. At the end of the treatment period, the total tumor burden present in *Slc28a1*^*−/−*^ mice was approximately double that in *Slc28a1*^*+/+*^ mice, as indicated by an increase in the average total bioluminescent flux on day 25 **(**Figs. 6B and 6C**)**. Furthermore, radiance values reached saturation at a fixed 1-s exposure 21 days post implantation in the vehicle control and dFdC-treated *Slc28a1*^*−/−*^ groups. However, full saturation was never achieved in the dFdC-treated *Slc28a1*^*+/+*^ group, even at day 25. The median overall survival of the vehicle control *Slc28a1*^*+/+*^ and *Slc28a1*^*−/−*^ mice was not significantly different (*p* < 0.02, log-rank test), with a mean overall survival of 23.5 and 24 days, respectively. However, the median overall survival of dFdC-treated *Slc28a1*^*−/−*^ mice was significantly (*p* < 0.02, log-rank test) shorter than that of dFdC-treated *Slc28a1*^*+/+*^ mice (39 days vs. > 50 days, respectively) **(**Fig. 6D**)**. All the *Slc28a1*^*−/−*^ mice died by day 45, whereas approximately 66% of the *Slc28a1*^*+/+*^ mice remained healthy at that time. These results highlight that reduced dFdC exposure in *Slc28a1*^*−/−*^ mice contributes to its reduced tumor efficacy and overall mouse survival, further supporting the role of CNT1 in renal reabsorption and retention of nucleosides and nucleoside drugs.

Since *Slc28a1*^*−/−*^ mice exhibit increased excretion of dFdC through renal elimination, we further increased the dose of dFdC administered to *Slc28a1*^*−/−*^ mice to achieve an equivalent drug exposure (calculated AUC) observed in *Slc28a1*^*+/+*^ mice receiving an intravenous dose of 25 mg/kg dFdC biweekly. *Slc28a1*^*−/−*^ mice receiving 25 mg/kg dFdC and *Slc28a1*^*−/−*^ mice receiving the adjusted dose of 37 mg/kg dFdC were dosed biweekly after orthoptic implantation of pancreatic cancer cells, and survival analysis was repeated until the mice succumbed to the disease or reached the exclusion criteria (Fig. 6E). The median overall survival of the *Slc28a1*^*−/−*^ mice in the 37 mg/kg treatment group was significantly higher (*p* < 0.02, log-rank test) than those in the 25 mg/kg treatment group, with a mean overall survival of 42 and 36 days in the former and latter groups, respectively (Fig. 6E). No adverse effects such as increases in serum liver enzymes and tumor-corrected body weight measurements were apparent with increased dosage of dFdC in *Slc28a1*^*−/−*^ mice. These results provide the proof-of-concept that reduced dFdC systemic exposure in *Slc28a1*^*−/−*^ mice due to urinary loss can be overcome by administering a higher dose.

## Discussion

By generating a KO mouse model and examining the alterations in the plasma and urinary metabolomes, we identified the essential role of CNT1 in renal salvage of endogenous pyrimidine nucleosides. By assessing the pharmacokinetics of the CNT1 cargo dFdC, we found that *Slc28a1*^*−/−*^ mouse plasma was less exposed to this chemotherapeutic due to increased urinary clearance. By evaluating survival and treatment outcomes in a mouse model of pancreatic cancer, we identified that CNT1 deficiency led to reduced antitumor drug efficacy, resulting in accelerated mortality. Finally, chemotherapeutic dose optimization compensated for the urinary loss of dFdC and restored survival in CNT1 null mice with no major adverse effects. Together, these findings illuminate the role CNT1 plays in renal retention of endogenous and synthetic pyrimidine nucleosides in murine kidneys and sheds light onto the unexpected therapeutic outcomes that could ensue due to factors such as *SLC28A1* gene mutations, *SLC28A1* genetic polymorphisms, *SLC28A1* transcriptional regulation (via factors such as HNF-4α), and *SLC28A1*-mediated nucleoside drug-drug interactions in patients.

CNT1’s role in the renal absorption of endogenous nucleosides was readily evident due to the increased excretion of several endogenous pyrimidine nucleosides in the urine of *Slc28a1*^*−/−*^mice under starvation. Loss of pyrimidine nucleosides through the urine of *Slc28a1*^*−/−*^ mice could create an imbalance in the cellular nucleotide pools and homeostasis at a normal physiological state, but we did not observe any major phenotype changes in *Slc28a1*^*−/−*^ mice fed at ad libitum, including in those mouse tissues that normally express high levels of CNT1. This is likely due to possible compensation through alterations in the purine metabolic pathways as observed in both *Slc28a1*^*−/−*^ plasma and urine metabolomes at a regular fed state. *Slc28a1*^*−/−*^ mice also had a similar lifespan compared to littermate WT controls. Nucleosides and nucleoside signaling mechanisms are known to play significant roles in mouse reproduction^57^, but the loss of CNT1 did not cause reduced fertility or fecundity or any visible birth defects in *Slc28a1*^*−/−*^ newborn mice. Together, these findings suggest that there is adequate compensation in mice even with the complete absence of the concentrative pyrimidine nucleoside transport activity mediated by CNT1. Only mild renal impairment with high urinary excretion of stress hormone (cortisol) levels was observed, which could be a direct consequence of the increased renal elimination of pyrimidine nucleosides, but this did not cause any observable long-term impacts on mouse health or survival. CNT1 KO mice were anemic but to a lesser extent than ENT3 KO mice, perhaps due to adequate bone marrow compensation, such as that seen with the increased turnover of regenerative erythrocyte precursors, including reticulocytes. Taken together, these results demonstrate the successful generation of a viable and fertile CNT1 KO mouse model with signs of pyrimidinuria.

The involvement of CNT1 in the renal reabsorption of pyrimidine (deoxy)nucleosides in murine kidneys especially during starvation recapitulates the observed trend of increased urinary excretion of nucleosides observed in humans with *SLC28A1* mutations^27,28^. These similarities suggest a possible conserved function for CNT1 in the renal reabsorption of pyrimidine nucleosides in mammalian kidneys. Importantly, the current study also sheds light onto several new insights into CNT1’s renal handling of nucleosides in a normal (i.e., well-fed) physiological state. First, our findings suggest an increase in the elimination of pyrimidine (deoxy)nucleosides with increased conversion of (deoxy)nucleosides to their corresponding (deoxy)nucleobases or conjugated metabolites and an increase in the excretion of amino acids and their derivatives, which is a characteristic of *Slc28a1*^−/−^ mouse urine. Second, the increased turnover of purine metabolism (both anabolic and catabolic) products in both *Slc28a1*^−/−^ mouse urine and plasma suggest that, in addition to the increased urinary excretion of pyrimidine nucleosides, the de novo purine metabolism pathways are activated in CNT1-deficient mice. Third, evidence from both the urine and plasma analyses showed that the pathways associated with several amino acid metabolism including those that act as building blocks for synthesis of purine nucleosides were activated. Altogether, these findings suggest that increased urinary excretion of pyrimidine (deoxy)nucleosides from *Slc28a1*^*−/−*^ mice is accompanied by putative compensatory alterations in purine (deoxy)nucleoside synthesis and degradation pathways along with changes in amino acid metabolism. Imbalances in pyrimidine and purine concentrations are associated with several diverse clinical manifestations, including neurological, immunological, hematological, and renal impairments^7,9^. However, the lack of such alterations in *Slc28a1*^*−/−*^ mice at all ages examined suggests that the changes in purine metabolism in *Slc28a1*^*−/−*^ mouse tissues is likely a compensatory mechanism to restore the balance between endogenous pyrimidine and purine concentrations to preserve *Slc28a1*^*−/−*^ mouse health.

CNT1 transports numerous FDA-approved nucleoside drugs used as frontline anticancer therapies (cytarabine, dFdC, clofarabine, fludarabine, capecitabine, 5-flurouridine, 5-fluro-2′-deoxyuridine etc.)^58–61^; therefore, CNT1 is likely a determinate of chemotherapeutic drug efficacy in patients^62^. dFdC is utilized as a probe compound in this study because it is used as both a primary and secondary agent for the treatment of numerous solid tumors, including pancreatic, breast, bladder, gallbladder, liver, testicular and ovarian tumors. The discovery of the increased excretion of endogenous pyrimidine nucleosides from *Slc28a1*^−/−^ mice prompted us to examine a putative role for CNT1 in the pharmacokinetics and disposition of dFdC. Like endogenous pyrimidine nucleosides, *Slc28a1*^*−/−*^ mice exhibited substantially increased urinary excretion of dFdC along with decreased systemic concentrations of dFdC, leading to its compromised therapeutic efficacy. In addition to dFdC, the excretion of the dFdC metabolite dFdU, another CNT1 cargo, was substantially increased in the urine of *Slc28a1*^*−/−*^ mice. These results suggest that CNT1 is a crucial determinate of dFdC efficacy in vivo that controls anticancer fluoropyrimidines drug exposure. Our findings also suggest that any functional alterations in CNT1 would significantly decrease treatment efficacy by compromising drug-induced mechanisms of growth control. dFdC, either alone or in combination with paclitaxel albumin-bound particles (Abraxane), is used as a frontline regimen for pancreatic cancer chemotherapy; however, its limited benefits on treatment and survival outcomes are of significant concern. In addition, dFdC is used as primary or secondary lines of treatment in several other solid tumors. It is possible that variations in CNT1 functionality may be a contributing factor to the poor efficacy of nucleoside analogs, as some patients with CNT1 deficiencies (e.g., due to renal diseases, CNT polymorphisms) may simply eliminate more drug in the urine, making less drug available for action on tumor tissues. Chemotherapeutic dose adjustments are frequently made in the clinic to accommodate patient conditions such as renal and hepatic impairments as well as to make up for patient variation in drug response due to factors such as mutations and polymorphisms to drug response genes. For instance, the doses of certain nucleoside drugs, such as 5’-fluorouracil and capecitabine, are routinely adjusted based on the functional status of dihydropyrimidine dehydrogenase (DPD)^63,64^ as DPD mediates 80% of the 5’-fluorouracil elimination through hepatic metabolism and determines 5’-fluorouracil exposure and efficacy^65,66^. Our current findings suggest that such adjustments to chemotherapeutic dosage based on prospective evaluation of CNT1 expression and renal functional status may be a potentially valuable strategy to optimize treatment outcomes in patients undergoing pyrimidine nucleoside analog therapies. However, further evaluation of the clinical translation of these findings are warranted.

In addition to fluoropyrimidine anticancer agents, CNT1 also transports several DNA methyltransferase inhibitors (e.g., 5-azacytidine, decitabine, zebularine), which are currently used to treat various leukemias, such as acute myeloid leukemia and chronic lymphocytic leukemia^67,68^ Moreover, recent studies have demonstrated significantly reduced expression levels of CNT1 in leukemic tissues, which may further worsen the predictability of the outcome of these drugs^30^. Additionally, CNT1 transports a wide range of antiviral (anti-HIV, anti-hepatitis B/C, anti-herpes) nucleoside drugs (e.g., zidovudine, lamivudine, zalcitabine, stavudine, emtricitabine, trifluridine, etc.) and agents used in the treatment of steroid-resistant nephrotic syndromes (e.g., mizoribine)^69^ Several reports have shown that these drugs exhibit significant pharmacokinetic variability in patients. Many of these drugs are also orally administered, and interestingly, CNT1 is highly expressed in the parts of the small intestine where absorption occurs, which perhaps contributes further to the oral absorption of nucleosides^22^. However, little is known about the practicable consequences of CNT1 functional alterations on pharmacokinetic variability with nucleoside-based therapies due to the intestinal effects of CNT1 in vivo, which would be of interest to study in the future. CNT mutations in humans that result in the urinary loss of nucleosides are beginning to be characterized. In addition, dysregulation of nucleoside homeostasis is associated with multiple nucleoside metabolic disorders, including Lesch-Nyhan syndrome, hereditary xanthinuria, orotic aciduria, and Von Gierke’s disease^9^. These conditions are routinely treated with oral uridine therapy and enzyme replacement therapy^70^. However, treatment efficacy is poor due to limited compound bioavailability^9,70^, which may be due to variability in CNT1 functionality in absorptive tissues, such as that demonstrated in this study. Currently, there are a total of 44 known *SLC28A1* single nucleotide polymorphisms (SNPs) that result in amino acid changes in hCNT1 as well as identified alternative splice variants that could potentially alter CNT1 function by either activating or inactivating nucleoside transport activity^35,71^. Therefore, the functional status of these SNPs and dose normalization are important considerations when dosing patients with pyrimidine nucleoside analog drugs.

In summary, we discovered a critical role for CNT1 in the renal reabsorption of endogenous and synthetic pyrimidine nucleosides that has clinical ramifications for the prediction and optimization of nucleoside analog efficacy. We anticipate that the CNT1 KO mouse model will be useful for further evaluating the role of CNT1 in nucleoside drug-drug interactions, comprehending CNT1 genetic polymorphisms, and understanding and treating CNT1-related inborn errors of metabolism.

## Methods

### Chemicals, reagents, and antibodies

Gemcitabine hydrochloride (dFdC), dFdC-^13^C,^15^N_2_ hydrochloride, 5′-deoxy-5′-fluorouridine (dFUR), dFdC triphosphate (dFdC-TP), and cytidine-^13^C,^15^N_2_ triphosphate were obtained from Toronto Research Chemicals (North York, ON, Canada). 2′,2′-Difluorodeoxyuridine (dFdU), tetrahydrouridine (THU), and d-luciferin were obtained from Millipore-Sigma (Burlington, MA, USA), Calbiochem (San Diego, CA, USA), and Gold Biotechnology (St. Louis, MO, USA), respectively. LC–MS grade methanol, acetonitrile, ammonium formate, formic acid, and ammonium hydroxide were obtained from Sigma-Aldrich (St. Louis, MO, USA). Distilled deionized water was prepared in-house using a Milli-Q water purification system (Millipore; Burlington, MA, USA) and was further filtered through a 0.22 µm filter before use. All other chemicals and reagents used in this study were of analytical reagent grade.

The DNeasy Blood & Tissue Kit (Qiagen; Valencia, CA, USA) was used to isolate genomic DNA, the RNeasy Mini Kit (Qiagen; Valencia, CA, USA) was used to isolate RNA, and the High-Capacity cDNA Reverse Transcription Kit (Thermo Fisher Scientific; Waltham, MA, USA) was used to convert RNA to cDNA for gene expression studies. The Pierce™ BCA Protein Assay Kit (Thermo Fisher Scientific; Waltham, MA, USA) was used to quantify protein levels before Western blotting analysis. Western blotting analysis was performed using an mCNT1 (Alomone Labs; ANT-061, 1:500) primary antibody with GAPDH (CST 97166, 1:5000) as a loading control. Rabbit (Bethyl; A120-201P, 1:5,000) and mouse (Bethyl; A90-116P, 1:5,000) secondary antibodies were used, and proteins were visualized using the SuperSignalTM West Pico PLUS Chemiluminescent Substrate (Thermo Fisher Scientific; Waltham, MA, USA) on a ChemiDoc MP Imaging System **(**Bio-Rad Laboratories; Hercules, CA, USA).

### Generation of Slc28a1^−/−^ mice

*Slc28a1*^*−/−*^ mice were generated by CRISPR/Cas9 technology at the Genetically Engineered Mouse Modeling Core (GEMMC) of the Ohio State University Comprehensive Cancer Center (OSUCCC) following an approved IACUC protocol. Briefly, single guide (sg) RNA sequences targeting *Slc28a1* were designed using the Benchling design tool^72^ (www.benchling.com). The chosen guide RNA sequences with the protospacer adjacent motif (PAM) in parentheses were as follows: 5′-CAGCTGAAGAGCCTAGCACA(TGG)−3′. The designed single strand (ss) DNA oligo of 150 nucleotides lacked the starting codon and the two nucleotides of the second codon residue [ATGGC] for use as a HDR template. In addition, a g.AG > CT mutation was introduced to disrupt a specific restriction site (AcuI) for genotyping purposes. The ssDNA sequence was as follows: 5′-TTTCTCTCTCCTGGCCCTCCTTTCGGCTGTGGACCCTCTGTGACATCTTTGTCTTTCAGCTGACTAGCCTAGCACAGACGACACACCGAGGCAACGAGAGTCCATTTCCCTCACACCTGTGGCCCATGGCCTGGAGAACATGGGGGCCGA-3′. Mouse C57BL/6NTac zygotes (Taconic; Rensselaer, NY) were injected with a mix of Cas9 nuclease (Thermo Fisher Scientific; Waltham, MA) (50 ng/µL final concentration), sgRNA (Millipore-Sigma; Burlington, MA) (1.5 µM final concentration) and ssDNA (Integrated DNA Technologies; Coralville, IA) (100 ng/µL final concentration). Genomic DNA was extracted from mouse tail clippings and used to detect the presence of *Slc28a1* mutations by PCR, restriction digestion, and sequencing in potential founder animals. To minimize the potential presence of off-target mutations, *Slc28a1*^*−/−*^ mice were backcrossed to C57BL/6NTac WT animals for three generations before breeding the homozygous animals used for subsequent experiments.

### Mouse husbandry and genotyping

All animal procedures were performed according to protocols approved by the Ohio State University (OSU) IACUC. A heterozygous breeding strategy was utilized to generate *Slc28a1*^*+/+*^, *Slc28a1*^*+/-*^, and *Slc28a1*^−/−^ littermates. Third generation (F3) *Slc28a1*^*+/−*^ mice were crossed to produce F4 *Slc28a1*^*+/+*^, *Slc28a1*^*+/-*^, and *Slc28a1*^−/−^ cohorts, and subsequent experiments were conducted on 8–20-week-old male and female *Slc28a1*^*+/+*^ and *Slc28a1*^−/−^ mice obtained through subsequent breeding. For genotyping, tail clippings were obtained from 3-week-old mice, and DNA was isolated using a Qiagen DNeasy Blood & Tissue Kit. Genotyping was performed by PCR using the following *Slc28a1* primers: forward 5′-CTCTCCCACTCTCCCCTTCT-3′ and reverse 5′-CCATAGCTGCCAGTCAAGC-3′. The PCR cycling conditions were as follows: initial denaturation at 98 °C for 30 s; 32 cycles at 98 °C for 10 s, 64 °C for 30 s, and 72 °C for 20 s; and a final extension at 72 °C for 1 min using Phusion High-Fidelity DNA Polymerase. Primers used for genotyping amplified a 402 bp region for *Slc28a1*^*+/+*^ and a 397 bp region for *Slc28a1*^−/−^ mice. After digestion with the AcuI (Eco571) restriction enzyme (Thermo Fisher Scientific, Waltham, MA) following the manufacturer’s protocol, the *Slc28a1*^*+/+*^ amplicon produced two bands at 149 bp and 253 bp. In contrast, the mutant amplicon remained undigested after the AcuI restriction digestion reaction. All mice were maintained at an ambient temperature of 20–22 °C and humidity 40–60% with a 12-h light/dark cycle and were given free access to standard rodent chow and water. Mice were euthanatized by carbon dioxide asphyxiation.

### Gene expression analysis

Total RNA was extracted from cells using an RNeasy Mini Kit (Qiagen, Valencia, CA) following the manufacturer’s protocol, and the RNA quality and quantity were measured using a NanoDrop^TM^ 2000 spectrophotometer (Thermo Fisher Scientific; Waltham, MA). Quantitative PCR (qPCR) detection of the *Slc28a1* gene was obtained after reverse transcription of 1 µg of total RNA using a High-Capacity cDNA Reverse Transcription Kit (Thermo Fisher Scientific; Waltham, MA). qPCR was performed using TaqMan^TM^ Fast Advanced Master Mix (Thermo Fisher Scientific; Waltham, MA) with a sequence-specific primers/probe set for *Slc28a1* (Thermo Fisher Scientific; Waltham, MA; assay ID: Mm01315355_m1). Similarly, sequence-specific qPCR primer sets (Integrated DNA Technologies, Inc., NC) were used for expression analysis of *Slc28a2, Slc28a3, Slc29a1*, and *Slc29a2* in *Slc28a1*^−/−^ and WT mice tissues (kidney, spleen, and pancreas). qPCR was performed using Fast SYBR™ Green Master Mix (Applied Biosystems™ #4385610; Thermo Fisher Scientific; Waltham, MA). The primer sequence details are:

*mSlc28a2*      F*:* 5′ CCAGGATGGAGATGTGGAAAT 3′

     R*:* 5′ TTCTAGGCCAACAGAGCATAAG 3′

*mSlc28a3*      F: 5′ AAACCAGCACAGGGTACATAG 3′

     R:5′ CTCACAGCACAGAGTGGAAA 3′

*mSlc29a1*      F: 5′ GGACAGGTATAAGGCAGTATGG 3′

     R: 5′ TCCAGGCGGTTTGTGAAATA 3′

*mSlc29a2*      F: 5′ CTGCCCTCCTGACTACATTTC 3′

     R: 5′ GTGTCCTAAGCAAGACCTACAG 3′

The ∆∆Ct method was used to determine the expression levels of the genes under investigation by normalizing the Ct values to *GAPDH* followed by the WT control. Each gene was amplified independently, and all experiments were performed in triplicate.

### Immunohistochemistry

Formalin-fixed paraffin-embedded tissue sections were prepared from the kidneys of *Slc28a1*^*+/+*^ and *Slc28a1*^*−/−*^ mice. The paraffin was removed from the tissue sections by baking at 60 °C for 1 h and two treatments with xylene for 10 min per wash. Next, the sections were rehydrated by submergence in a gradient series of ethanol solutions. Antigen retrieval was performed by microwave treatment for 15 min at 10% power in citrate buffer, pH 6. A DAB staining kit was obtained from Abcam (ab64264), and the manufacturer’s staining protocol was followed. The anti-CNT1 primary antibody was diluted 1:200, and sections were incubated with this primary antibody overnight at 4 °C. Harris hematoxylin solution was used to perform nuclear costaining. Images were captured at ×20 magnification with a Vectra 2.0 imaging system (Perkin Elmer).

### Drug preparation and administration

A dFdC stock solution (10 mg/ml) was prepared in saline (0.9% NaCl) no more than 1 h before administration. A bolus dose of dFdC (50 mg/kg) or an equivalent volume of vehicle control (0.9% NaCl) was administered intravenously via the tail vein before the pharmacokinetic studies. Biweekly intravenous administrations of dFdC (25 or 37 mg/kg) via retro-orbital injection was performed for dFdC efficacy and survival studies in mice with orthoptic pancreatic tumors.

### Blood and urine collection

After dFdC administration, blood and urine samples were collected at various time points using a combination of submandibular, retro-orbital, and cardiac puncture collection methods following a previously published protocol^73^. To collect blood, a 5 mm lancet was used to apply pressure to the submandibular vein and release blood flow. For retro-orbital collection, mice were anesthetized with 3% isoflurane in oxygen, and the blood was collected directly into heparinized capillary tubes by applying slight pressure to the medial canthus of the eye. Cardiac puncture was performed as the final collection method by first euthanizing the mice in a CO_2_ chamber and immediately performing posterior cardiac puncture by inserting a 27 G needle with a 1 ml syringe attachment into the heart. Once blood collection was completed, gentle pressure was applied to the blood collection area using gauze to stop the bleeding. Thirty microliters of blood was collected into heparinized capillary tubes at each time point, transferred into prelabeled 0.5 ml microcentrifuge tubes containing THU (10 µg/ml; final concentration), and immediately processed and snap-frozen following procedures outlined in the “Gemcitabine plasma pharmacokinetics” methods section. To collect urine, mice were placed in a plastic container and scruffed at each time point; the resulting urine was collected via pipette, mixed with THU (10 µg/ml; final concentration), and stored at −80 °C for subsequent analysis.

### Urinalysis

Spot urine collected from *Slc28a1*^+/+^ and *Slc28a1*^−/−^ male and female mice were resolved using SDS-PAGE. Albumin standards (0.5, 1.0 and 2.0 µg bovine serum albumin [BSA]) were resolved on the same 8% gels, which was then stained with Coomassie Brilliant Blue G-250 (Alfa Aesar, Tewksbury, MA) to visualize bands. The albumin concentration was determined by densitometry from the standard curves using Image J software (National Institutes of Health, Bethesda, MD). Urinary Creatinine was measured using the Enzymatic Creatinine Test Kit (Diazyme, Poway, CA). Creatinine standards were run on the same plate to build a standard curve to determine the absolute creatinine concentrations in the urine samples and a control creatinine amount was also run to ensure accuracy of the test.

### Western blotting

Lysates from mouse tissues were made in TNE buffer supplemented with protease and phosphatase inhibitors. After sonication, a clarifying spin (5000 × *g* for 10 min at 4 °C), and protein quantification with a Pierce™ BCA Protein Assay Kit (Thermo Fisher Scientific; Waltham, MA), 20 µg of total cell lysate was separated by SDS-PAGE on an 8% polyacrylamide gel using a Bio-Rad gel electrophoresis system following the manufacturer’s protocol. Separated proteins were transferred onto PVDF membranes using the Bio-Rad Trans-Blot Turbo Transfer System. Western blotting analysis was performed using an mCNT1 (Alomone Labs; ANT-061, 1:500) primary antibody with GAPDH (CST 97166, 1:5000) as a loading control. Rabbit (Bethyl; A120-201P, 1:5000) and mouse (Bethyl; A90-116P, 1:5000) secondary antibodies were used, and proteins were visualized using the SuperSignalTM West Pico PLUS Chemiluminescent Substrate (Thermo Fisher Scientific; Waltham, MA) on a ChemiDoc MP Imaging System from Bio-Rad. Uncropped blots are provided in the Data Source file.

### Targeted metabolomics analysis of nucleosides and nucleoside derivatives

To prepare samples for targeted LC–MS/MS analysis, cytosine, uracil, thymine, adenine, guanine, cytidine, uridine, thymidine, adenosine, guanosine, deoxycytidine, deoxyuridine, deoxyadenosine, and deoxyguanosine nucleotide standards were prepared in 50:50 H_2_O:MeOH, and internal standards of 13C8-guanine, 15N5-deoxyguanosine, and 13C10-guanosine were prepared each at a concentration of 500 ng/ml. Standard solutions of all nucleotide targets were serially diluted to generate calibration solutions at concentrations of 0.0, 0.0001, 0.001, 0.01, 0.1, 1.0, and 10 µg/ml. Urine samples were prepared by adding 20 µL of urine solution to 80 µL of 50:50 H_2_O:MeOH containing internal standard, vortexing and centrifuging at 20,000 × *g*; then, 50 µL aliquots were placed into glass vials, dried in a SpeedVac and reconstituted in 5% MeOH with 0.1% formic acid for injection.

Samples were quantified using a heated electrospray ionization source (HESI) on a Thermo Scientific TQS Quantiva triple quadrupole mass spectrometer, and separation was achieved using a Thermo Scientific Ultimate 3000 HPLC equipped with an Agilent 120 SB-C18 reversed-phase column (2 × 100 mm, 2.7 µm particle size) maintained at 40 °C. Samples (5 µL) were injected and separated at a flow rate of 200 µL/min with a solvent system of 100% H_2_O containing 0.1% formic acid as solvent A and 100% MeOH containing 0.1% formic acid as solvent B. The gradient program was as follows: 5% solvent B, increasing linearly to 10% solvent B at 2 min, followed by an increase to 50% solvent B by 8.0 min, reaching 100% solvent B at 8.5 min, holding at 100% solvent B until 10 min, returning to 5% solvent B at minute 12, and equilibration at 100% solvent B until minute 15. For nucleobases and nucleosides, the monitored transitions are listed in Table 1. with their respective mass/charge for the precursor and product ions and collision energies. For all experiments, the capillary voltage was set to 4.5 kV with a capillary temperature of 350 °C, a vaporizer temperature of 100 °C, a sheath gas flow of 12, an auxiliary gas flow of 13, and a 1.6 sweep gas. For selected reaction monitoring (SRM) mode, Q1 and Q3 were set to 0.7 full width at half maximum (FWHM) resolution, a cycle time of 0.8 s, and a CID gas pressure of 1.5 mTorr.

**Table 1:** The mass/charge for the precursor and product ions and collision energies of nucleobases and nucleosides analyzed by targeted metabolomics analysis

Compound	Precursor (*m*/*z*)	Product (*m*/*z*)	Collision energy (V)	RF Lens (V)
Cytosine	112.05	52	28.88	65
Cytosine	112.05	94.982	18.73	65
Uracil	113.035	70	17.3	60
Uracil	113.035	96	17.38	60
Thymine	127.05	54.071	24.71	59
Thymine	127.05	110.071	16.84	59
Adenine	136.062	92.054	30.27	77
Adenine	136.062	119.054	24.17	77
Guanine	152.057	110.071	21.13	70
Guanine	152.057	135.071	17.97	70
HL-Guanine	162.057	117.125	22.94	84
HL-Guanine	162.057	144.196	19.66	84
Deoxycytidine	228.098	95.06	34.61	30
Deoxycytidine	228.098	111.994	19.11	30
Deoxyuridine	229.082	113.143	13.21	40
Deoxyuridine	229.082	117.125	11.07	40
Thymidine	243.098	110.101	31.5	36
Thymidine	243.098	127.071	12.08	36
Cytidine	244.093	112.143	10.27	45
Cytidine	244.093	127.167	13.05	45
Uridine	245.077	113.083	13.68	43
Uridine	245.077	133.982	11.11	43
Deoxyadenosine	252.109	119.042	41.77	59
Deoxyadenosine	252.109	136.065	17.3	59
Adenosine	268.104	136.065	17.55	65
Adenosine	268.104	152.012	16.12	65
Deoxyguanosine	268.104	136.083	23.7	69
Deoxyguanosine	268.104	152.125	14.01	69
HL-Deoxyguanosine	273.104	139.125	32.72	66
HL-Deoxyguanosine	273.104	157.143	13	66
Guanosine	284.099	151.101	23.24	77
Guanosine	284.099	152.143	18.31	77
HL-Guanosine	299.099	162.173	14.65	74
HL-Guanosine	299.099	281.137	14.65	74

### Untargeted metabolomics

To prepare samples for untargeted LC–MS/MS analysis, *Slc28a1*^*+/+*^ and *Slc28a1*^−/−^ mouse urine and plasma samples were protein precipitated by spiking 20 μL of each sample with 80 μL of ice-cold methanol followed by incubation for 30 min at −20 °C and centrifugation 20,000 × *g*. The supernatant was then aliquoted into LC vials for subsequent analysis. Pooled QC samples were prepared by mixing an equivalent portion of four samples from each group. Prior to MS detection, samples were separated on a Poroshell 120 SB-C18 column (2 × 100 mm, 2.7 μm particle size) with an Agilent 1290 Infinity UHPLC system. The system consisted of solvent A (H_2_O with 0.1% formic acid) and solvent B (100% methanol) and was operated at a flow rate of 200 μL/min with the following gradient program: 2% solvent B for 3 min, a linear increase to 45% B at 11.5 min, then at 90% B from minute 13 to minute 20, back to 2% B at 25 min and equilibration with 2% B until minute 30. Five microliters of each sample were injected, and all analyses were performed on an Agilent 6545 quadrupole time-of-flight mass spectrometer in sensitivity mode and positive polarity with electrospray ionization (ESI). MS/MS data-dependent analysis was performed in which the top 5 ions were selected within a 30 second exclusion window, and all data sets were collected in centroid mode. For feature selection, including database comparison and statistical processing, samples were analyzed with Progenesis QI 3.0. ANOVA *p* value scores between the *Slc28a1*^*+/+*^ and *Slc28a1*^*−/−*^mice were calculated, a cutoff *p* value of <0.05 was selected, and database matching was performed using the Human Metabolome Database, selecting for adducts (M + H, M + Na, M + 2H, and 2M + H) with less than 10 ppm mass error. Initially, raw files were converted to mzXML and uploaded and aligned in Progenesis QI 3.0 using pooled QC samples where features were filtered to exclude those features with % CV higher than 30 %. Normalization was also performed in Progenesis using a global scaling factor between each samples full set of detected compounds and their ratio to the pooled QC in a log scale. For annotation of features, only those features with an abundance of greater than 1000 counts were accepted. Additionally, only features containing MSMS data and theoretical fragmentation scores of 20% in Progenesis Metascope using the latest sdf file of HMDB were tentatively identified. For urine analysis, 1932 features over 1000 abundance, <0.05 *p* value, and <30% CV for pooled samples were found and 204 were tentatively identified. For plasma, 323 features were found at the same cut-offs and 57 were tentatively identified. Pathway analysis was conducted and heatmaps were constructed using the MS peak pathway finder and statistical analysis features of MetaboAnalyst 4.0 from those features identified using Progenesis QI^46,47^.

### Metabolite network analysis

The *mummichog* algorithm and an adapted GSEA method were used to predict pathway activities from our untargeted metabolomics data in MetaboAnalyst 4.0. Metabolomics data, including the *m/z* features, *p* values, and statistical scores, were uploaded into the MS Peaks to Pathways module. Parameters for upload included a mass accuracy of 5 ppm, positive ion mode, and a *p* value ≤ 0.05. KEGG metabolic pathways for *Mus musculus* (mouse) were used in conjunction with a KEGG-style global metabolic network to allow visualization of the compounds within each pathway. The *mummichog* algorithm was then applied to generate mummichog pathway analysis plots with a global KEGG metabolic network (Fig. 4) following mathematical procedures described by Chong et al.^47^. The mummichog pathway analysis plots display all the matched pathways as circles, with the color and size of each circle corresponding to its *p* value and enrichment factor, respectively, where the enrichment factor is the ratio between the number of significant (*p* < 0.001) pathway hits and the expected number of pathway hits. Ranked pathways that were enriched were determined using a hypergeometric test to determine raw *p* values, and calculated *p* values were determined using a gamma distribution modeled with observed data.

### Gemcitabine plasma and urine pharmacokinetics

Single-dose pharmacokinetic plasma profiling was conducted in 8- to 12-week-old female *Slc28a1*^*+/+*^ and *Slc28a1*^−/−^ mice after i.v. administration of dFdC (50 mg/kg) suspended in saline (0.9% NaCl). Serial blood collection was performed following a previously published protocol^73^. Briefly, blood samples were collected into hepatized capillary tubes at seven time points (5, 10, 15, 30, 60, 120, and 240 min), mixed with THU (10 µg/ml), and centrifuged (1500 *× g* for 5 min), and the resulting plasma supernatant was immediately stored at −80 °C. Gemcitabine and dFdU plasma concentrations were measured using a modified version of our previously published UHPLC–MS/MS method for nucleosides^74^ (Figs. S6 and S7). Noncompartmental pharmacokinetic parameters (*C*_max_, *T*_max_, AUC, CL, *T*_1/2_, etc.) were calculated using WinNonlin 6.2 software (Pharsight; Mountain View, CA). The elimination rate constant (K_el_) was estimated from the slope of the terminal phase of the log plasma concentration-time curve fitted by the least-squares method, and the terminal half-life (T_1/2_) was calculated by 0.693/*K*_el_. The *C*_max_ and *T*_max_ were obtained from the concentration-time curve. The AUC was calculated according to the linear trapezoidal rule up to the last time point with a measurable concentration of the analyte of interest. Significant differences between *Slc28a1*^*+/+*^ and *Slc28a1*^−/−^ mouse pharmacokinetic parameters (*p* < 0.05) were evaluated with an unpaired two-sided Student’s *t*-test.

### UHPLC–MS/MS analysis and quantification of dFdC, dFdC-TP and dFdU in plasma

To prepare samples for LC–MS/MS analysis, *Slc28a1*^*+/+*^ and *Slc28a1*^*−/−*^ mouse plasma and urine samples were protein precipitated by spiking 10 µL of each sample with 90 µL of ice-cold methanol containing 50 ng/ml of each internal standard (^13^C,^15^N-dFdC, 5′-deoxy-5′-fluorouridine, and ^13^C,^15^N-cytidine triphosphate) as previously reported^75,76^, incubated for 30 min at −20 °C, and centrifuged at 20,000 × *g* for 25 min. The resulting supernatant was evaporated to dryness in a SpeedVac, and the remaining residue was reconstituted in mobile phase A for subsequent analysis. For tissue analysis, 10 mg of tissue was collected, and dFdC, dFdU, and dFdC-TP extraction was achieved by homogenizing the tissue in ice-cold methanol (50% v/v) containing THU (25 µg/ml) and 50 ng/ml each internal standard in a Precellys 24 tissue homogenizer for a final tissue homogenate concentration of 0.05 mg/µL. Subsequent processing was identical to that for the plasma and urine samples.

Stock solutions of dFdC and dFdC-TP (1 mg/ml) were made in water, while dFdU was dissolved in methanol. Appropriate working solutions were used to spike blank urine, plasma, and tissue samples derived from untreated *Slc28a1*^*+/+*^ mice as described above to generate calibration standards in the following concentration ranges: 10–2500 ng/ml (0.2–50 ng/mg) for dFdC and dFdC-TP and 20–5000 ng/ml (0.4–100 ng/mg) for dFdU. Quality control samples were prepared in the same way to give the appropriate concentrations.

Previously reported chromatography conditions were adapted and coupled to the Waters Triple Quadrupole Mass Spectrometer with an Acquity UHPLC for the analysis of dFdC, dFdU, and dFdC-TP. Briefly, the analysis was performed on an Imtakt Schzero SM C18 column, 2.1 × 100 mm, 3.0 µm particle size. The eluents used consisted of mobile phase A consisting of 50 mM ammonium formate:50 mM ammonium hydroxide (9:1, *v/v*) (pH = 8.6) and mobile phase B consisting of 50 mM ammonium formate:50 mM ammonium hydroxide (9:1, v/v):ACN (80:20, *v/v*) (pH = 8.6). A gradient program was used for the separation and identification of dFdC and its metabolites at a flow rate of 0.5 ml/min. The program was initiated with 100% mobile phase A from 0–4 min, increased to 50% mobile phase A from 4–20 min, remained at 50% mobile phase A from 30–32 min, and increased to 100% mobile phase A from 32–40 min. The injection volume was 10 μL, and the autosampler temperature was set at 4 °C throughout the analysis.

The Waters Triple Quadrupole Mass Spectrometer with an ESI source was operated in negative and positive ion mode at a spray voltage of 2.5 kV, capillary temperature of 150 °C, and vaporizer temperature of 250 °C. Compound optimization was performed manually using the MassLynx 4.1 Software AutoTune Wizard (Waters, Milford, MA) by infusion into the mass spectrometer using a T-connector. The ion transitions at m/z 264.03 ↔ 112.04, 504.00 ↔ 326.07, 263.00 ↔ 202.13, 267.0 ↔ 368.1, 483.0 ↔ 385.0, and 522.3 ↔ 424.0 were used for dFdC, dFdU, dFdC-TP, dFdC-IS, dFdU-IS, and dFdC-TP-IS, respectively, in multiple reaction monitor (MRM) mode. Collision energy values were optimized to 22–28% for these transitions. Analyze concentrations were determined and analyzed using MassLynx Mass Spectrometry Software (Waters; Milford, MA).

### UHPLC–MS/MS analysis and quantification of dFdC and difluorodeoxyuridine in mouse urine

Gemcitabine and dFdU concentrations in mouse urine were measured in a liquid chromatography–tandem mass spectrometry (LC–MS/MS) method. THU was added to the mouse urine samples upon collection and frozen until analysis. The samples were thawed on ice then diluted 50-fold and 200-fold with 1.25 µg/mL THU in deionized water. Five microliters of the diluted samples were transferred to a 96-well autosampler plate then 10 µL of internal standard mixture was added and mixed. The internal standard mixture contained 125 ng/mL dFdC-^13^C,^15^N_2_ hydrochloride and 1000 ng/mL 5′-deoxy-5′-fluorouridine in 97:3 1 mM ammonium acetate pH 6.8:acetonitrile (v/v). The plate was then transferred to the 4 °C autosampler for analysis.

The chromatographic separation was performed on a Thermo Accucore aQ 2.6 µm, 100 ×2.1 mm column at 30 °C using a gradient with a 10 µL injection volume. The mobiles phases were 1 mM ammonium acetate in deionized water pH 6.8 and acetonitrile. The total run time was 5.5 min. The dFdC and dFdU chromatography was based on Vainchtein, et al.^77^. The LC–MS/MS analysis was conducted on a Thermo Vanquish UHPLC with a TSQ Quantiva mass spectrometer equipped with an electrospray ionization source. Gemcitabine and its internal standard, dFdC-^13^C,^15^N_2_ hydrochloride, were measured by selected reaction monitoring (SRM) in positive mode at *m/z* of 264.138 → 112 and 267.13 → 115.071, respectively. For quantitation of dFdU, the *m/z* transitions monitored in negative polarity were 263.05 → 220.054 for dFdU and 245.042 → 129 for the internal standard, 5′-deoxy-5′-fluorouridine. The linear ranges established were 10–10,000 ng/mL for dFdC and 20–20,000 ng/mL for dFdU.

### Orthotopic implantation of KPC cells into the pancreases of syngeneic recipients

Pancreatic tumor cells derived from a *PDX-1-CRE, LSL-KRas*^G12D^, LSL-Trp53^−/−^ (KPC) genetically engineered mouse model (GEMM) transfected with enhanced firefly luciferase (KPC-LUC) were implanted into the pancreases of *Slc28a1*^*+/+*^ and *Slc28a1*^*−/−*^ mice following our previously published protocol^56,78^. Briefly, KPC-LUC cells were cultured in DMEM (without sodium pyruvate) supplemented with 10% fetal bovine serum (FBS) until 90% confluence, trypsinized, centrifuged, and resuspended at a concentration of 2.5 × 10^5^ cells/50 µL in a sterile mixture containing 1:1 (*v/v*) HBSS and serum-free Matrigel Matrix (Corning; Corning, NY). Twelve *Slc28a1*^*+/+*^ female mice and 12 *Slc28a1*^*−/−*^ female mice between 8 and 12 weeks of age were orthotopically injected with the KPC-LUC cell suspension into the tail region of the pancreas^78^. Mice received meloxicam injections (5 mg/kg) every 24 h for 3 days post implantation, were left to recover and were monitored for one week before tumor visualization and treatment initiation.

### Luciferase imaging and tumor burden quantification

Tumor growth was visualized by the bioluminescent signal produced after intraperitoneal injection of D-luciferin (150 mg/mouse; Gold Biotechnology, Inc.; St. Louis, MO) using an IVIS Lumina II Imaging System (Caliper Life Sciences; Waltham, MA) after anesthetization with 1.5–2.5% isoflurane (Baxter, Deerfield, IL, USA). All mice were imaged on days 7, 14, 21, and 25 post implantations using a fixed image exposure time of 1 s 5 min after D-luciferin administration. All acquired images reported luminescent radiance (p/sec/cm^2^/sr) on a fixed scale, with a minimum of 7.15 × 10^8^ and a maximum of 2.22 × 10^10^. The total tumor burden was calculated by subtracting the total bioluminescent flux produced by the entire mouse from the background bioluminescent signal using Living Image software 4.8.0.

### Statistical analysis

All statistical analyses were performed using GraphPad 9.0.

### Reporting summary

Further information on research design is available in the Nature Portfolio Reporting Summary linked to this article.

## Supplementary information

Supplementary Information

Description of Additional Supplementary Files

Supplementary Data 1

Supplementary Data 2

Reporting Summary

## Data Availability

The data sets generated during and/or analyzed during the current study are all available within the article and its Supplementary Information files. A reporting summary for this article is available as a Supplementary Information file. Metabolomics data have been deposited in the EMBL-EBI MetaboLights database (10.1093/nar/gks1004. PubMed PMID: 23109552) with the study identifier MTBLS1768. Source data are provided with this paper.

## Source data

Source Data
