# Author Correction: Increased renal elimination of endogenous and synthetic pyrimidine nucleosides in concentrative nucleoside transporter 1 deficient mice

**DOI:** 10.1038/s41467-023-39187-w

**Published:** 2023-06-07

**Authors:** Avinash K. Persaud, Matthew C. Bernier, Michael A. Massey, Shipra Agrawal, Tejinder Kaur, Debasis Nayak, Zhiliang Xie, Brenna Weadick, Ruchika Raj, Kasey Hill, Nicole Abbott, Arnav Joshi, Nadeen Anabtawi, Claire Bryant, Arpad Somogyi, Zobeida Cruz-Monserrate, Foued Amari, Vincenzo Coppola, Alex Sparreboom, Sharyn D. Baker, Jashvant D. Unadkat, Mitch A. Phelps, Rajgopal Govindarajan

**Affiliations:** 1 Division of Pharmaceutics & Pharmacology, College of Pharmacy, The Ohio State University, Columbus, OH 43210 USA; 2 Campus Chemical Instrument Center Mass Spectrometry and Proteomics Facility, The Ohio State University, Columbus, OH 43210 USA; 3 The Center for Life Sciences Education, College of Arts and Sciences, The Ohio State University, Columbus, OH 43210 USA; 4 Division of Nephrology & Hypertension, Renaissance School of Medicine, Stony Brook University, Stony Brook, NY 11794 USA; 5 Pharmacoanalytic Shared Resource (PhASR), The Ohio State University, Columbus, OH 43205 USA; 6 Center for Clinical & Translational Research, Nationwide Children’s Hospital, Columbus, OH 43210 USA; 7 Division of Gastroenterology, Hepatology, and Nutrition, College of Medicine, The Ohio State University, Columbus, OH 43210 USA; 8 Genetically Engineered Mouse Modeling Core, Ohio State University Comprehensive Cancer Center, The Ohio State University, Columbus, OH 43210 USA; 9 Department of Cancer Biology and Genetics, College of Medicine, The Ohio State University, Columbus, OH 43210 USA; 10 Department of Pharmaceutics, College of Pharmacy, University of Washington, Seattle, WA 98195 USA; 11 Translational Therapeutics, Ohio State University Comprehensive Cancer Center, Ohio State University, Columbus, OH 43210 USA

**Keywords:** Carrier proteins, Cancer therapeutic resistance, CRISPR-Cas9 genome editing

Correction to: *Nature Communications* 10.1038/s41467-023-38789-8, published online 01 June 2023

In this article, the author name Sharyn D. Baker was incorrectly written as Sharyn D. Bakfer. The original article has been corrected.

