# Supplementary material for: Increased renal elimination of endogenous and synthetic pyrimidine nucleosides in concentrative nucleoside transporter 1 deficient mice
Source: Nat Commun. 2023 Jun 1;14:3175. doi: 10.1038/s41467-023-38789-8 (PMC10235067; doi:10.1038/s41467-023-38789-8)
Supplement: Supplementary file 1 — Supplementary Information [file 41467_2023_38789_MOESM1_ESM.pdf]

Increased renal elimination of endogenous and synthetic pyrimidine nucleosides in concentrative nucleoside transporter 1 deficient mice

Persaud et al.

Supplementary Information

Supplementary Figures 1-7

Supplementary Tables 1-5

Supplementary Figure 1

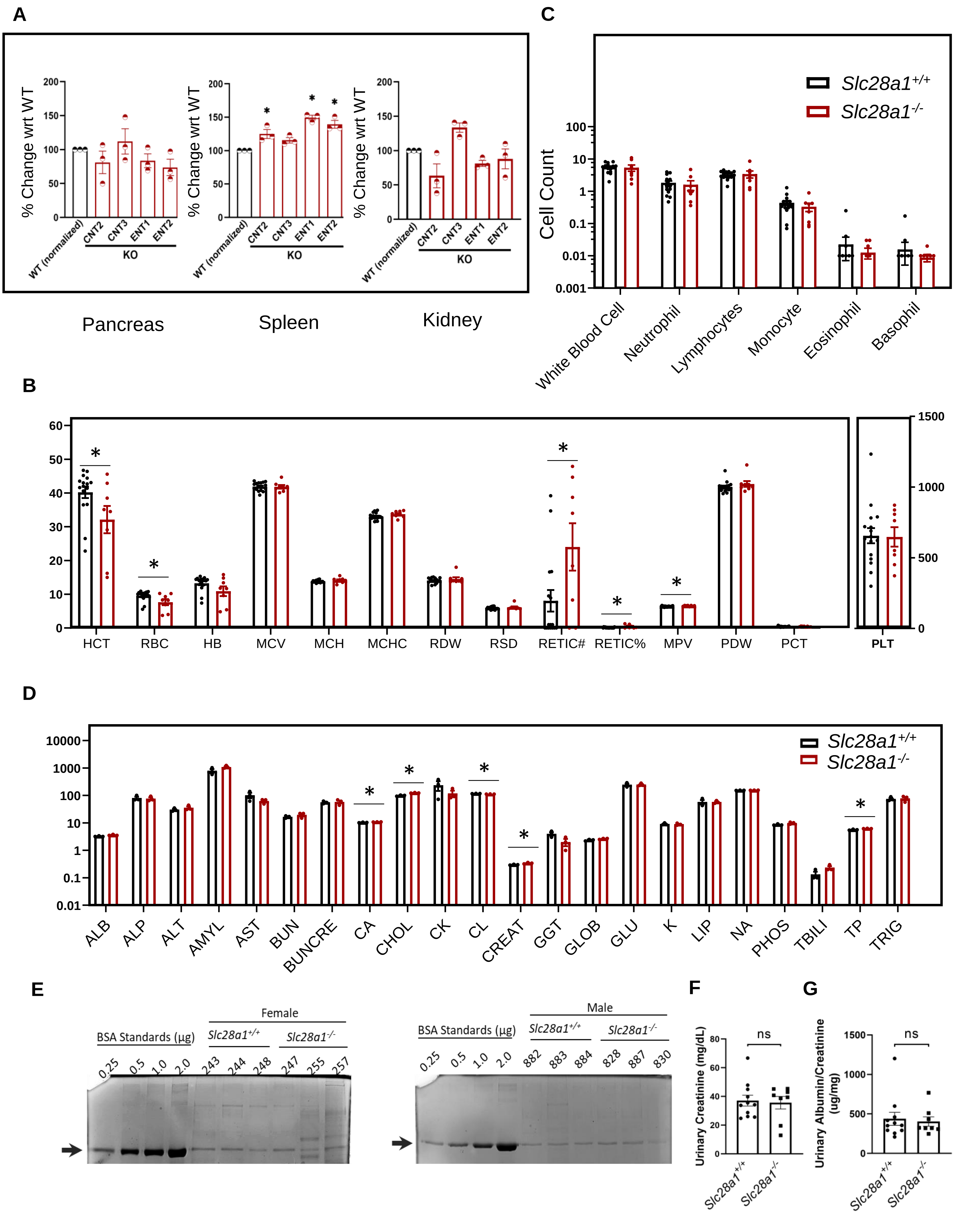

Supplementary Figure 1. **Characterization of CNT1-null mice for blood and urinary parameters. (A)** Relative gene expression in *Slc28a1*$^{-/-}$ mouse organs normalized to GAPDH and *Slc28a1*$^{+/+}$ control displayed as a percent change (2-ΔΔCt). Data represent mean ± SEM (n=3 mice/group mean ± SEM, *$p < 0.05$ by two-tailed t-test). **(B)** Red blood cell and platelet parameters, including HCT (hematocrit; %), RBC (red blood cell count; 10^6/uL), HB (hemoglobin; g/dL), MCV (mean cell volume; fL), MCH (mean corpuscular hemoglobin; pg), MCHC (mean cell hemoglobin; pg), RDW (red cell distribution width; %), RSD (red cell (erythrocyte) standard deviation), RETIC%# (reticulocyte number; 10^9/L), RETIC# (reticulocyte %; 10^9/L), MVP (mean platelet volume; fL), PDW (platelet distribution width; %), PCT (plateletcrit; ug/L), and PLT (platelet count; 10^9/L) were identified in 12-week-old *Slc28a1*$^{-/-}$ *(black)* and *Slc28a1*$^{+/+}$ *(red)* mice. Data represent mean ± SEM (n=13 mice/group mean ± SEM, *$p < 0.05$ by two-tailed t-test). **(C)** White blood cell count, including neutrophil, lymphocyte, monocyte, eosinophil, and basophil counts, were identified in 12-week-old *Slc28a1*$^{-/-}$ *(black)* and *Slc28a1*$^{+/+}$ *(red)* mice (*n = 13,* mean ± SEM). Data represent mean ± SEM (n=13 mice/group mean ± SEM). **(D)** Clinical chemistry results, including ALB (albumin; g/dL), ALP (alkaline phosphatase; U/L), ALT (alanine transaminase; U/L), AMYL (amylase; U/L), AST (aspartate aminotransferase; U/L), BUN (blood urea nitrogen; mg/dL) BUNCRE (blood urea nitrogen:creatinine), CA (calcium;mg/dL), CHOL (cholesterol; mg/dL), CK (creatine kinase, U/L), CL (chloride; mmol/L), CREAT (creatinine; umol/L) were identified in 12-week-old *Slc28a1*$^{-/-}$ *(red)* and *Slc28a1*$^{+/+}$ *(black)* mice. Data represent mean ± SEM (n=3 mice/group mean ± SEM, *$p < 0.05$ by two-tailed t-test). **(E)** Albumin bands (~66 kDa) shown on representative urine samples resolved on a gel. Equal volumes of urine (5 µl) were resolved on SDS-PAGE from female and male *Slc28a1*$^{-/-}$ and *Slc28a1*$^{+/+}$ mice and stained (n=6 mice (3 males and 3 females)/group). BSA (bovine serum albumin) was resolved on the same gel at specified amounts to generate a standard curve to determine albumin concentration from *Slc28a1*$^{-/-}$ and *Slc28a1*$^{+/+}$ mice **(F)** Urinary creatinine was measured using the Diazyme creatinine assay and the data represent mean ± SEM (n=8 *Slc28a1*$^{-/-}$ mice; n=11 *Slc28a1*$^{+/+}$ mice/group, *$p < 0.05$ by two-tailed t-test) **(G)** Urinary Albumin/Creatinine ratio was determined and plotted. Data represent mean ± SEM (n= n=8 *Slc28a1*$^{-/-}$ mice; n=11 *Slc28a1*$^{+/+}$ mice/group, *$p < 0.05$ by two-tailed t-test)

Supplementary Figure 2

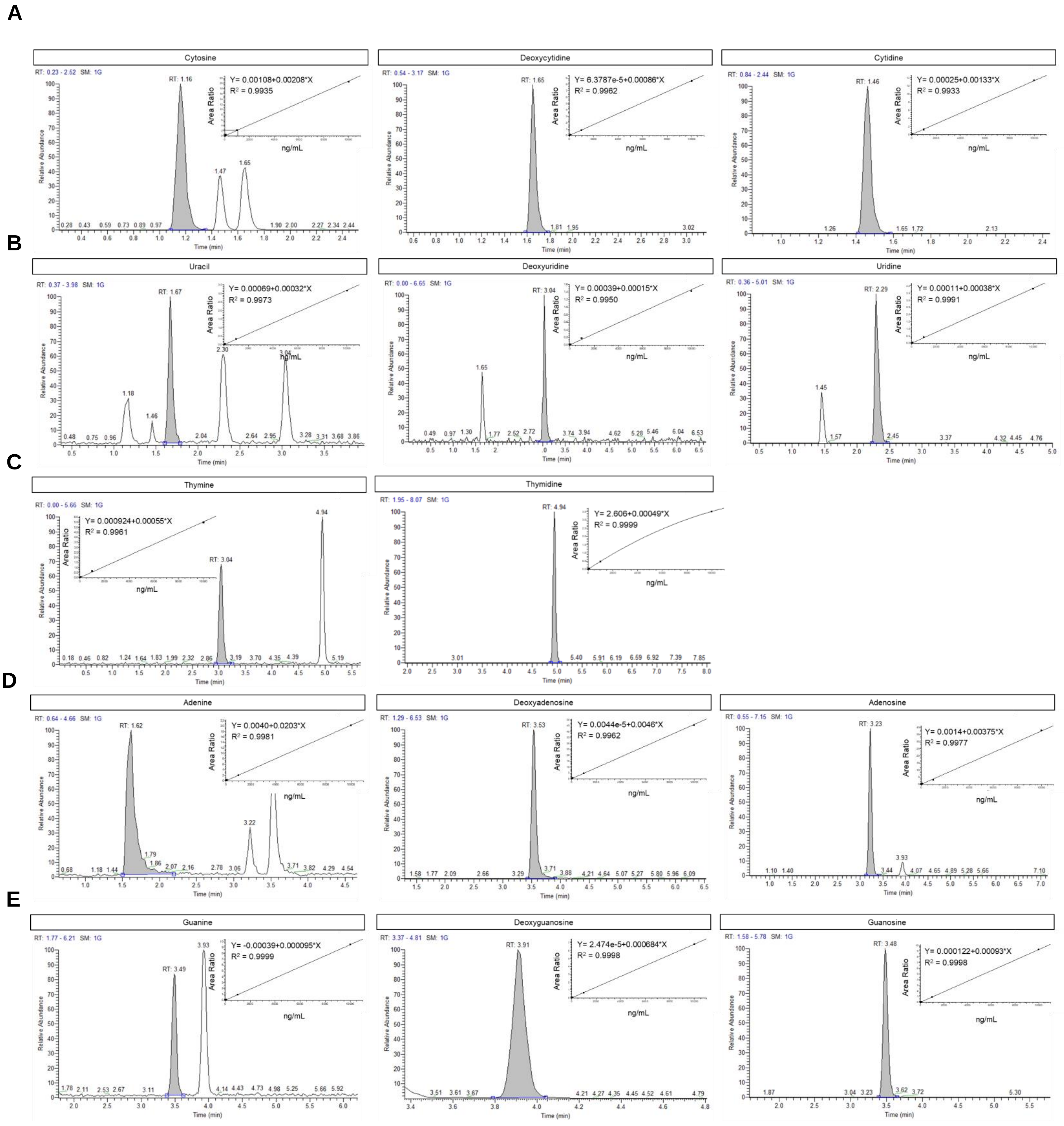

Supplementary Figure 2: Analyte chromatograms and standard curves from targeted LC-MC/MS analysis of *Slc28a1*$^{-/-}$ mice urine. (A) LC chromatographs and standard curves for cytosine and cytosine derivatives. **(B)** LC chromatographs and standard curves for uracil and uracil derivatives. **(C)** LC chromatographs and standard curves for thymine and thymine derivatives. **(D)** LC chromatographs and standard curves for adenine and adenine derivatives. **(E)** LC chromatographs and standard curves for guanine and guanine derivatives

Supplementary Figure 3

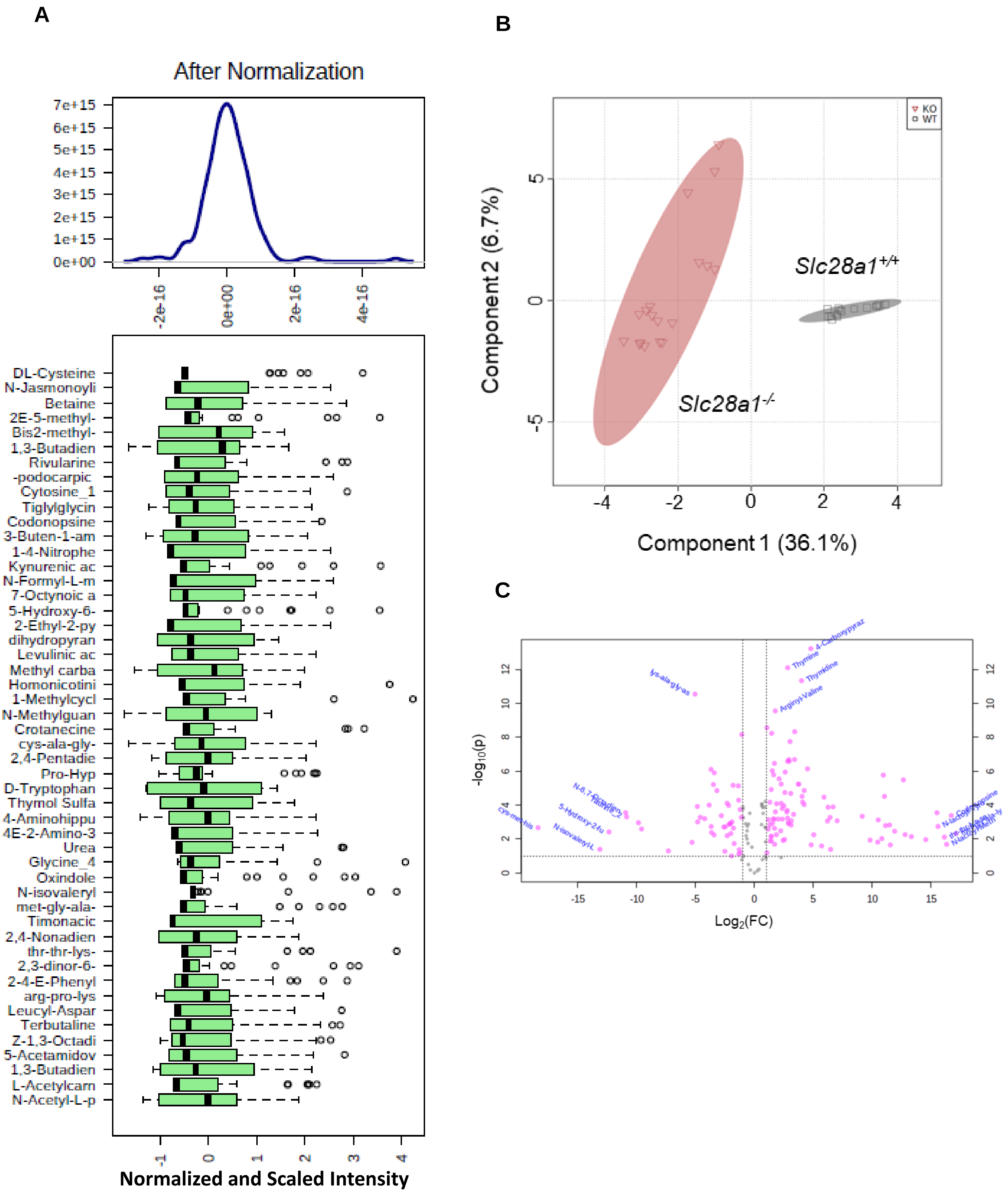

Supplementary Figure 3. Untargeted metabolomics normalization of *Slc28a1$^{-/-}$* *and Slc28a1$^{+/+}$* mouse urine. Untargeted metabolomics normalization of *Slc28a1*$^{-/-}$ and *Slc28a1*$^{+/+}$ mouse urine. **(A)** Box plots and kernel density plots after normalization. The boxplots show median, minima and maxima for at most 50 features due to space limit. The density plots are based on all samples. Selected methods: Row-wise normalization: N/A; Data transformation: N/A; Data scaling: Autoscaling. Analysis of differentially produced metabolites in *Slc28a1*$^{-/-}$ urine (n=6). Data are presented as mean values +/- SD. **(B)** Projection to latent structure-discriminant analysis (PLS-DA) score plot constructed based on metabolic profiles of *Slc28a1*$^{+/+}$ and *Slc28a1*$^{-/-}$ urine samples (black, *Slc28a1*$^{+/+}$; red, *Slc28a1*$^{-/-}$) (n = 6). **(C)** Volcano plots showing metabolite profiles of *Slc28a1*$^{+/+}$ compared with *Slc28a1*$^{-/-}$ urine samples. Dotted lines along x axis represent ± log2(2) fold change and dotted line along y axis represents –log10(0.1). p-values derived from two-sided t-test. pink, differential metabolites; grey, other metabolites.

Supplementary Figure 4

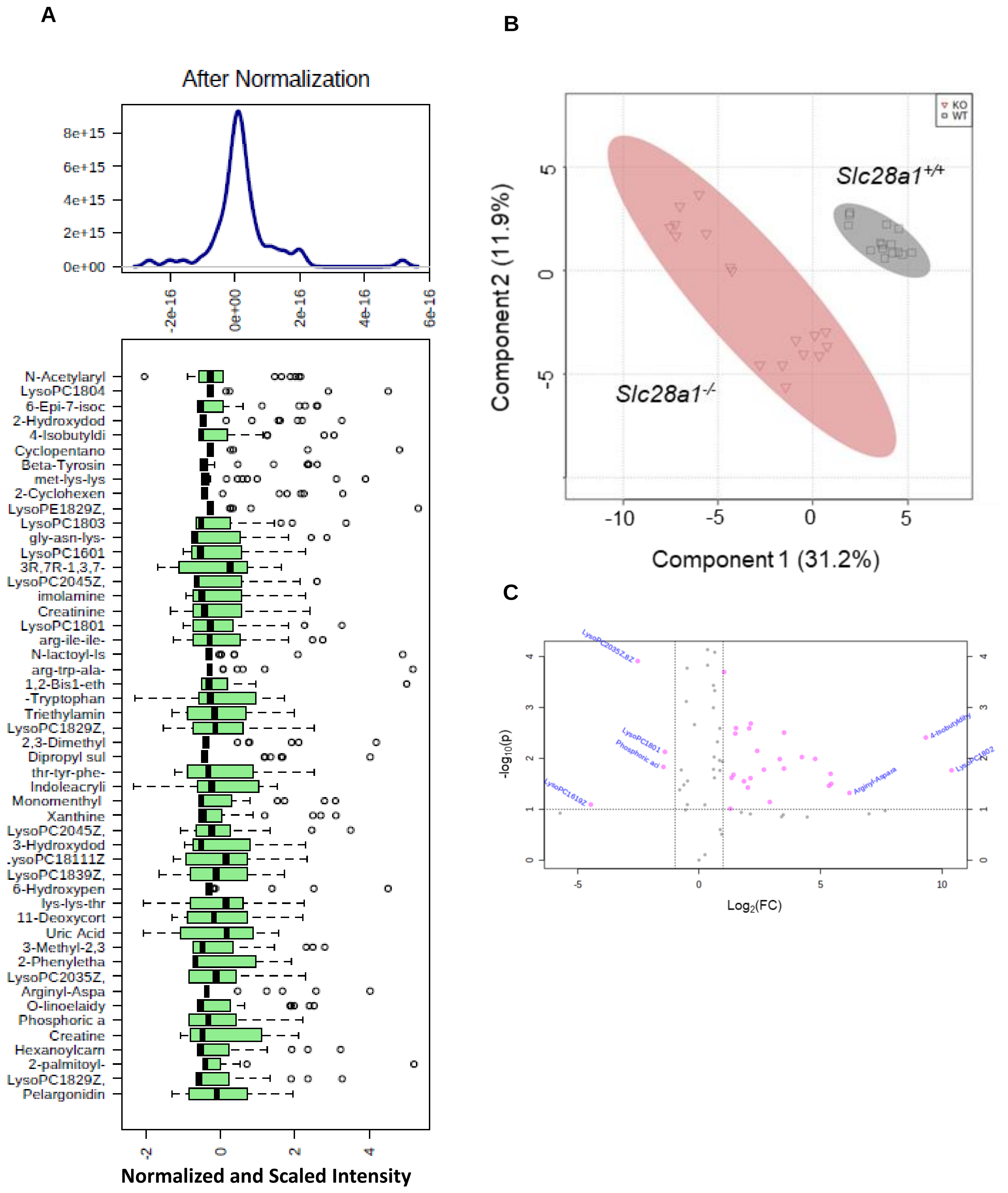

Supplementary Figure 4. Untargeted metabolomics normalization of *Slc28a1*$^{-/-}$ and *Slc28a1*$^{+/+}$ mouse plasma. Box plots and kernel density plots after normalization. **(A)** The boxplots show median, minima and maxima for at most 50 features due to space limit. The density plots are based on all samples. Selected methods: Data scaling: Autoscaling. Analysis of differentially produced metabolites in *Slc28a1*$^{-/-}$ plasma (n=6). Data are presented as mean values +/- SD. **(B)** Projection to latent structure-discriminant analysis (PLS-DA) score plot constructed based on metabolic profiles of *Slc28a1*$^{+/+}$ and *Slc28a1*$^{-/-}$ plasma samples (black, *Slc28a1*$^{+/+}$; red, *Slc28a1*$^{-/-}$) (n =6). **(C)** Volcano plots showing metabolite profiles of *Slc28a1*$^{+/+}$ compared with *Slc28a1*$^{-/-}$ plasma samples. Dotted lines along x axis represent ± log2(2) fold change and dotted line along y axis represents –log10(0.1). p-values derived from two-sided t-test. pink, differential metabolites; grey, other metabolites.

Supplementary Figure 5

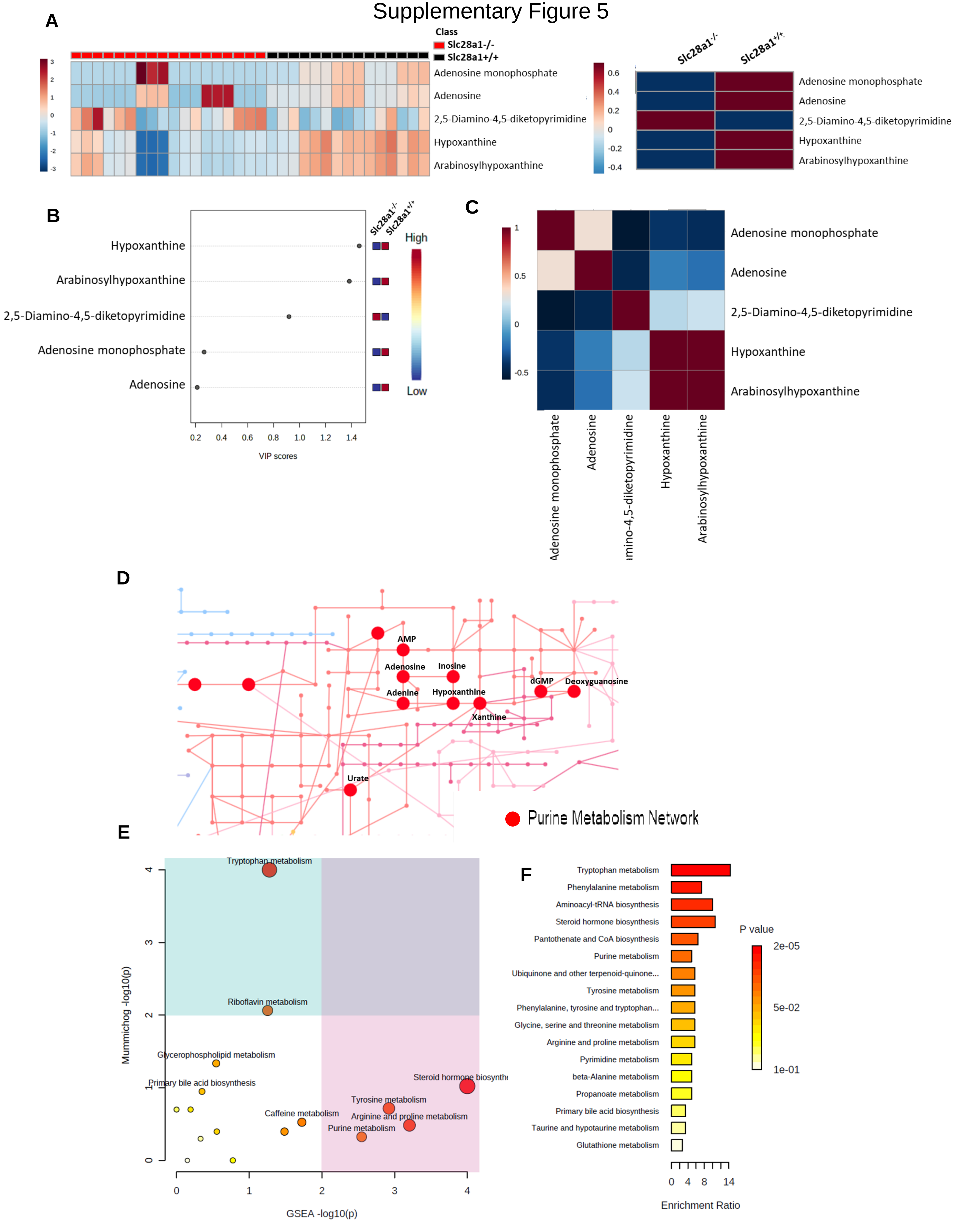

Supplementary Figure 5. Untargeted metabolomics analysis and metabolite pathway analysis of *Slc28a1*$^{-/-}$ mice plasma. **(A)** Heatmap illustrating hierarchical clustering of differential features (*left*) and the average abundances (*right*) for nucleoside derived metabolites detected across 5 *Slc28a1*$^{+/+}$ and 6 *Slc28a1*$^{-/-}$ mice plasma samples run in triplicate by mass spectrometry-based metabolomics. Data represent mean ng/ml ± SEM (n=12 mice/group mean ± SEM, *p < 0.05 (Progenesis ANOVA Scores). MS signal intensities for all heatmaps were clustered in two dimensions based on Euclidean distance (*row*, metabolites; *column*, samples). Colors indicate the metabolite abundances (*red*, high; *blue*, low). For identified metabolites, increased (*red*) or decreased (*blue*) fold change in *Slc28a1*$^{-/-}$ and corresponding p-value (*black*) indicated. **(B)** VIP (Variable Importance in Projection) Scores for annotated nucleoside derived features in partial Least Squares-discriminant Analysis (PLS-DA), **(C)** Correlation heatmap illustrating the overall correlation between different features. **(D)** Network visualization of the purine and pyrimidine metabolite networks with altered purine metabolites highlighted red and altered pyrimidine metabolites highlighted in blue for urine metabolomics data using Fisher's method of MS Peaks-to-Paths analysis, **(E)** the Mummichog and GSEA pathway Meta-analysis for MS Peaks to Paths combining the separate algorithms' p-values (*p < 0.05 by one-tailed hypergeometric test) , and **(F)** Quantitative enrichment analysis using the concentration table of the final annotated list of features for the untargeted differential analysis for *Slc28a1*$^{-/-}$ *vs Slc28a1*$^{-/-}$ mice urine using the HMDB codes for each feature and the KEGG library (*p < 0.05 by two-tailed Welch's t test).

Supplementary Figure 6

A

Gemcitabine (dFdC)

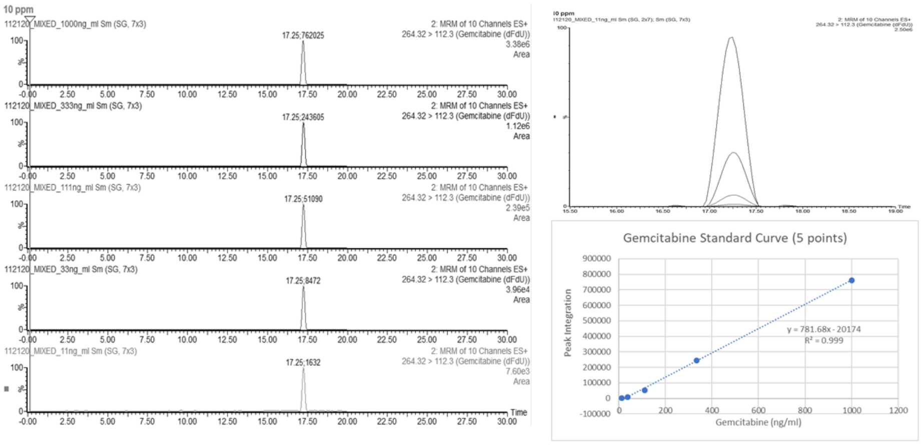

B

13C,15N-Gemcitabine (dFdC-IS)

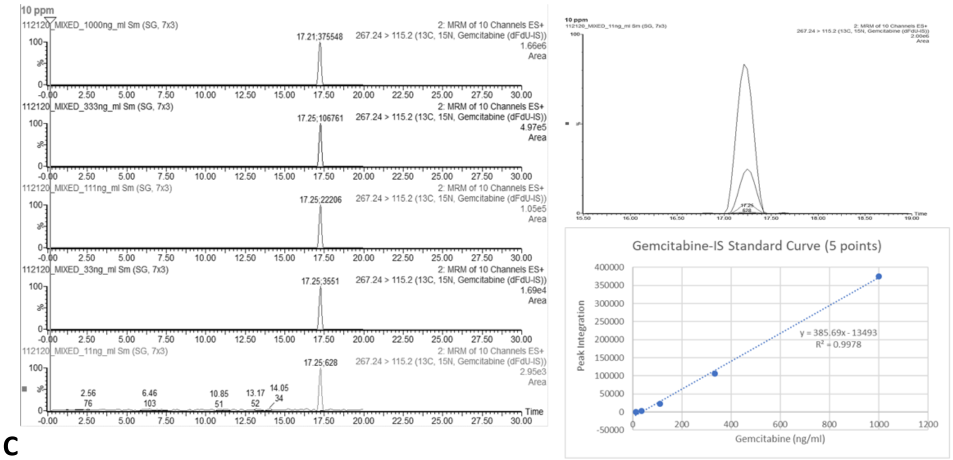

C

Gemcitabine Triphosphate (dFdC-TP)

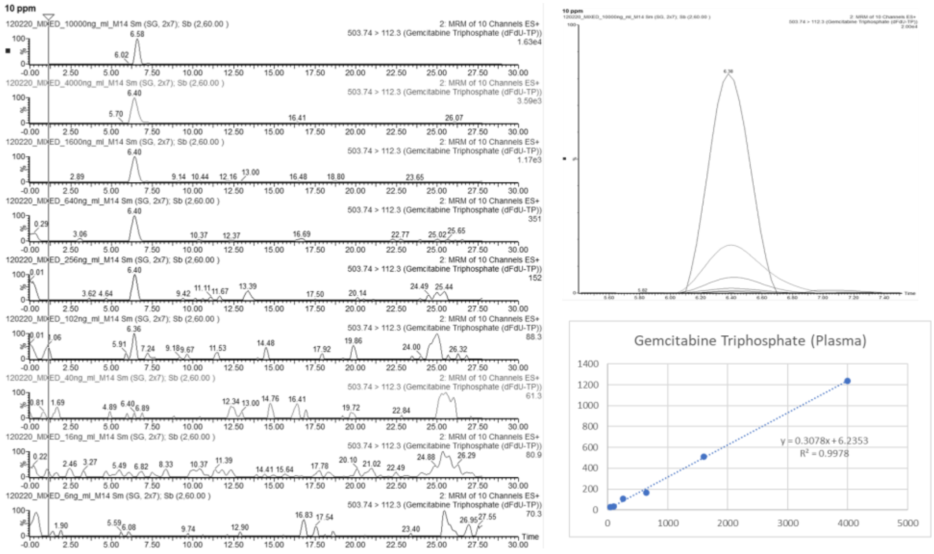

Supplementary Figure 6: Analyte chromatograms and standard curves for gemcitabine, $^{13}C,^{15}N$-gemcitabine and gemcitabine triphosphate LC-MC/MS analysis of *Slc28a1*$^{-/-}$ mouse plasma and urine. (A) LC chromatographs and standard curves for gemcitabine. **(B)** LC chromatographs and standard curves for $^{13}C,^{15}N$-gemcitabine. **C)** LC chromatographs and standard curves for gemcitabine triphosphate.

Supplementary Figure 7

A

2’,2’-Difluorodeoxyuridine (dFdU)

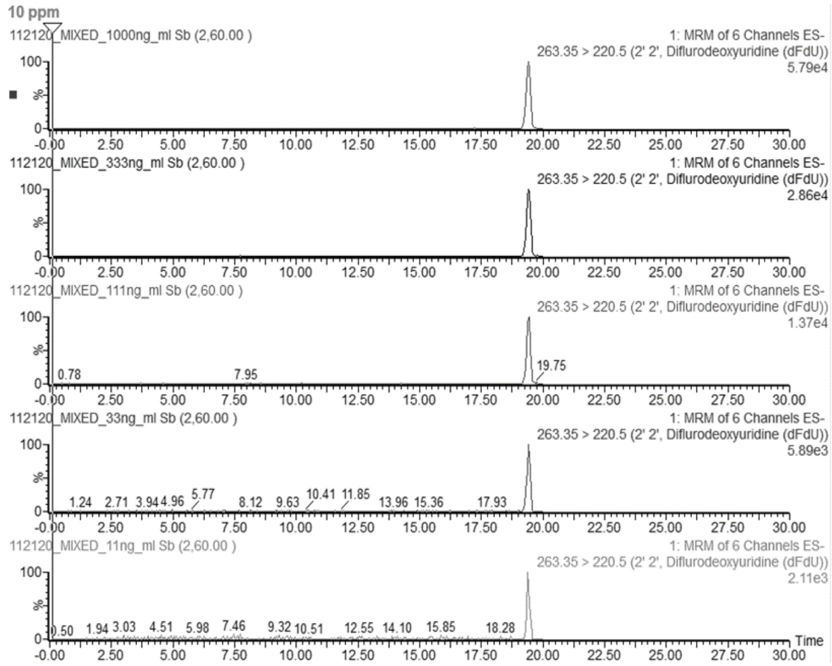

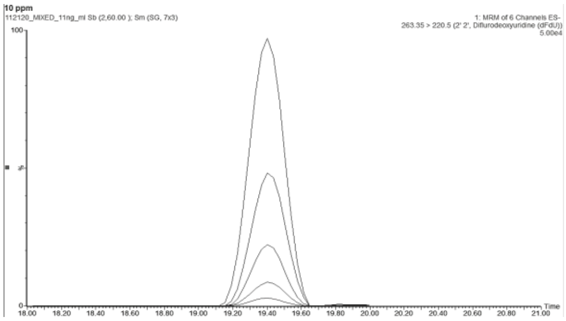

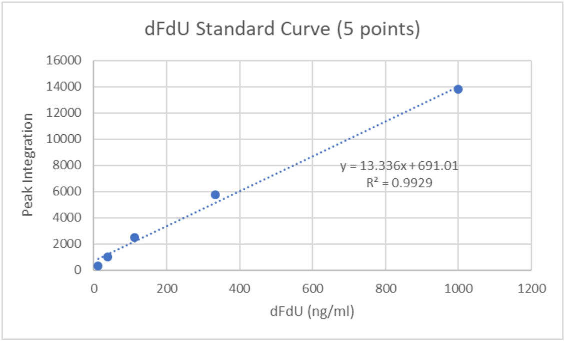

B

5’-Deoxy-5’-fluorouridine (dFdU-IS/dFUR)

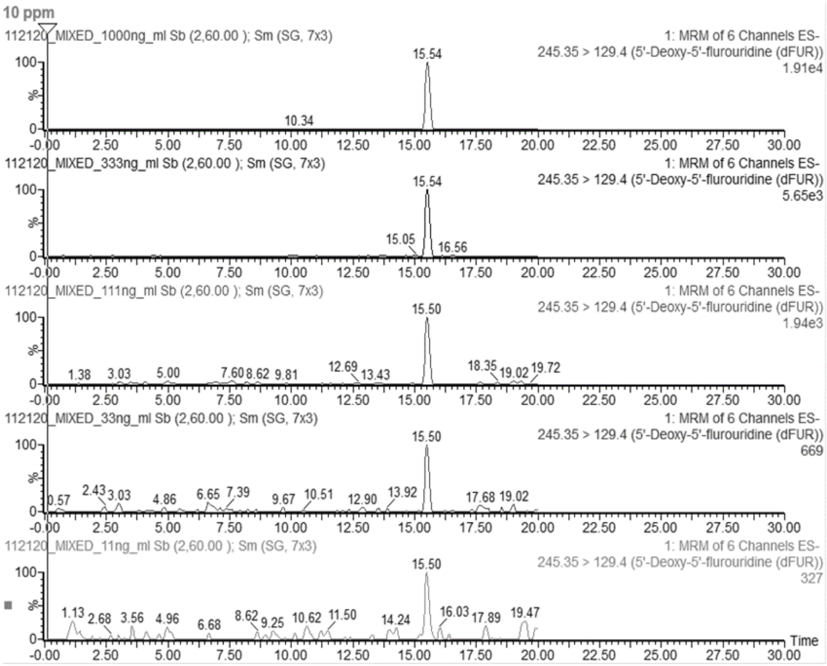

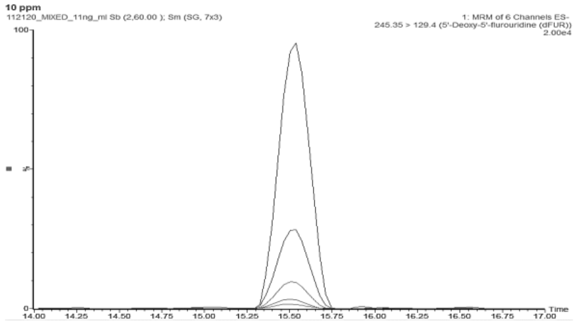

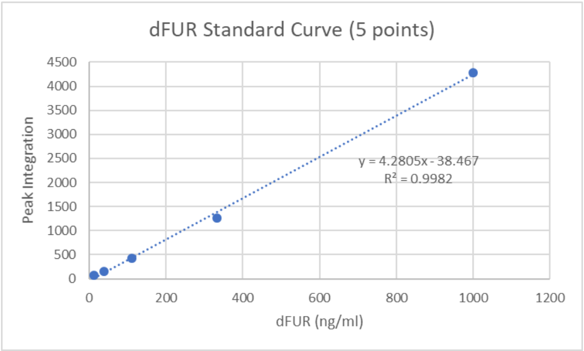

Supplementary Figure 7: Analyte chromatograms and standard curves for difluorodeoxyuridine (dFdU) and 2’,2’-difluorodeoxyuridine LC-MC/MS analysis of *Slc28a1*$^{-/-}$ mouse plasma and urine. (A) LC chromatographs and standard curves for 2’,2’-difluorodeoxyuridine (dFdU). **(B)** LC chromatographs and standard curves for 5’-deoxy-5’-fluorouridine (dFdU-IS/dFUR).

Supplementary Table 1

Sequence	PAM	Score	Gene	Chromosome	Strand	Position	Mismatches	On-target
CAGCTGAAGAGCCTAGCACA	TGG	100	ENSMUSG00000025726	chr7	1	81115537	0	TRUE
AAACTGAAAAGCCTAGCACA	TGG	1.726466667		chr12	-1	24845404	3	FALSE
CAACTGAAGTGCCTAGCACC	TGG	1.609602648		chr16	-1	44103759	3	FALSE
GAGCTGACGAGCCTAGCAGA	TGG	1.414893617		chr18	1	60631609	3	FALSE
CATCTGCACAGCCTAGCACA	GGG	1.099569865		chr5	1	147629355	3	FALSE
CAGATGGAGATCCTAGCACA	GGG	1.048360262		chr4	-1	14813088	3	FALSE
CCACTGTTGAGCCTAGCACA	AAG	0.99548667		chr13	1	34767268	4	FALSE
TAAATGCAGAGCCTAGCACA	CAG	0.971304808		chr18	-1	82105271	4	FALSE
CACATGGTGAGCCTAGCACA	TAG	0.963503163		chr8	-1	51318787	4	FALSE
CAGCAGAAGATCCTAGCACC	TAG	0.888336364		chr13	-1	34774121	3	FALSE
CAACCGAAGAGCCTTGCACA	CAG	0.885487125		chr13	1	33355321	3	FALSE
CAACCGAAGAGCCTTGCACA	CAG	0.885487125		chr13	1	33280691	3	FALSE
CTACTGAGGATCCTAGCACA	CGG	0.882129808	ENSMUSG00000040433	chr9	-1	96747360	4	FALSE
CAGCTCAATGGCCTAGCACA	CAG	0.852252022		chrX	-1	78584018	3	FALSE
CAGGCTAGGAGCCTAGCACA	GAG	0.832166988		chr6	1	31011924	4	FALSE
CTTCTGAAGTGCCTAGCACT	AAG	0.827636945		chr14	1	71249230	4	FALSE
AAGGGGAAGAGGCTAGCACA	CAG	0.807718894		chr11	1	75595817	4	FALSE
TCGCTGAAGTGTCTAGCACA	GAG	0.795213177	ENSMUSG00000036768	chr9	1	122988005	4	FALSE
CAGCTGCAGAGCCCAGCACA	TGG	0.721482463		chr4	1	126025882	2	FALSE
AACCTGAAGGGCCTAGGACA	GGG	0.711728078	ENSMUSG00000015599	chr17	1	46442935	4	FALSE
GGGCAGAAGAGCCTAGCAGA	GAG	0.65625		chr1	1	43061706	4	FALSE
TAGATGGAAAGCCTAGCACA	CAG	0.64358336		chrX	-1	139613110	4	FALSE
AGGCTGCAGAGCCTAGCACT	CGG	0.630210675		chr4	-1	152449818	4	FALSE
CCGATGAAGAGCCTACCACA	GAG	0.629672447	ENSMUSG00000068923	chr3	1	88746906	3	FALSE
CAGCTCCACAGCCTAGCACA	AGG	0.61264533		chr1	1	57628904	3	FALSE
CAGCTGCAAAGCCTAGCACC	AGG	0.608908085		chr13	-1	34251453	3	FALSE
AAGTTTGAGAGCCTAGCACA	GGG	0.600848342		chr15	-1	28691321	4	FALSE
TAGCAACAGAGCCTAGCACA	GGG	0.595983173		chr14	-1	66932363	4	FALSE
GAGCTTAGCAGCCTAGCACA	GAG	0.565191389		chr4	-1	62258767	4	FALSE
CACCAAGAGAGCCTAGCACA	GGG	0.560412872		chr10	1	70066802	4	FALSE
CAGGGAGAGAGCCTAGCACA	AGG	0.555501297		chr1	1	38185290	4	FALSE
CATGTGCAGAGGCTAGCACA	CAG	0.524608202		chr17	1	24919308	4	FALSE
CAGTTGAAGAGCATAGAACA	AGG	0.521345304	ENSMUSG00000092473	chr7	-1	23953311	3	FALSE
CTGCTGAAGAGCATAGCAGA	GAG	0.51816443		chr7	1	134264785	3	FALSE
CAACTGGGGAGGCTAGCACA	CAG	0.51544474		chr8	-1	118422678	4	FALSE
CAACTGGCGAGACTAGCACA	GGG	0.51544474		chr2	1	74464646	4	FALSE
GAACTGAAGAGCCAAGCACA	GAG	0.51406372		chr6	1	137957805	3	FALSE
AGGCTGAGGAGCCTTGCACA	CAG	0.50515873		chr17	-1	31434602	4	FALSE
GAGCTTAAGAGCCTAGCTCA	AGG	0.50403132		chr15	-1	80779446	3	FALSE
CCGCTGGAGGGGCTAGCACA	TGG	0.503449826	ENSMUSG00000049800	chr11	-1	20650425	4	FALSE
CAGCACAAGAGCCTTGCACA	CAG	0.500920325		chr16	-1	5850168	3	FALSE
TAGATGATGAGCCTGGCACA	GAG	0.494689119		chr8	1	104097301	4	FALSE
CTGCTTAAGCACCTAGCACA	GAG	0.494035319		chr19	1	40275506	4	FALSE
TAGCGGAGGAGCCTGGCACA	GAG	0.489615385		chr10	-1	41392477	4	FALSE
CAGCCAAGGATCCTAGCACA	TGG	0.48824426		chr14	1	80825040	4	FALSE
ATGATGAAGAGCCTGGCACA	GAG	0.48464467		chr2	-1	5140429	4	FALSE
TCGCTGAAGAATCTAGCACA	CAG	0.483968284		chr1	1	57825352	4	FALSE
AAAGTGAAGAGCCTGGCACA	TAG	0.473057035		chr4	-1	11673606	4	FALSE
GACCTGAAGATTCTAGCACA	CAG	0.472491321		chr14	1	113695173	4	FALSE
CCAGTGAAGAGCCTTGCACA	GAG	0.459211463		chr13	1	107721060	4	FALSE

Supplementary Table 1. Off-target effect summary for *Slc28a1* knockout mice generation. *Table displays gRNA sequence parameters used for CRISPR/Cas9 genome editing of *Slc28a1*.

Supplementary Table 2

Accepted Description	t.stat	p.value	minusLOG10(p)	FDR	Fold Change	log2(FC)	Frag. Score	Mass Error (ppm)	Isotope Similarity
N-Acetyl-S-allylcysteine	-30.8	8E-25	24.1	2E-22	3E-02	-5.1	41.6	5.0	92.6
hydroxyvalerylglycine	-25.5	2E-22	21.6	3E-20	1E-01	-2.8	41.6	5.2	95.6
Glutamylmethionine	-24.9	5E-22	21.3	4E-20	2E-02	-5.8	29.0	2.8	92.0
1-hydroxyhexanoylglycine	-23.7	2E-21	20.7	1E-19	2E-01	-2.4	42.6	4.1	93.7
Indole-3-acetic-acid-O-glucuronide	-22.3	1E-20	19.9	5E-19	3E-01	-1.7	62.3	2.4	94.0
2-Aminoheptanedioic acid	-22.3	1E-20	19.9	5E-19	3E-01	-1.8	58.6	4.9	96.5
Acetylcysteine	-20.1	2E-19	18.6	7E-18	2E-01	-2.2	22.3	4.9	91.0
2,4-Pentadienal	-20.0	3E-19	18.5	7E-18	4E-01	-1.3	25.8	9.8	98.8
Butane-1,1-diamine	-19.7	4E-19	18.4	9E-18	3E-01	-1.9	34.6	8.0	96.5
Thiazole, 4,5-dihydro-2,4,4-trimethyl-	-18.7	2E-18	17.7	4E-17	3E-04	-11.6	50.0	6.5	93.9
(8Z)-3-Hydroxydodec-8-enoylcarnitine	18.6	2E-18	17.7	4E-17	4E+00	2.2	73.2	8.9	93.7
Tiglylglycine	-18.5	3E-18	17.6	5E-17	4E-01	-1.4	36.7	7.3	97.8
2-Aminohippuric acid	-18.3	4E-18	17.4	6E-17	5E-01	-1.1	67.0	6.7	96.9
2-hydroxyhexanoylglycine	-17.4	1E-17	16.8	2E-16	4E-01	-1.2	42.4	4.7	96.4
4-hydroxyhexanoylglycine	-17.4	2E-17	16.8	2E-16	3E-01	-1.6	33.0	5.2	96.6
N-Acetyl-S-(3-hydroxypropyl)cysteine	-17.3	2E-17	16.8	2E-16	4E-01	-1.5	62.2	4.0	92.0
3-Hydroxybutyrylcarnitine	-17.1	3E-17	16.6	3E-16	2E-02	-6.0	38.7	3.0	93.3
5-Acetamidovalerate	16.8	4E-17	16.4	5E-16	3E+00	1.7	54.8	5.8	99.1
N-Acetyl-DL-homocysteine	-16.4	8E-17	16.1	9E-16	1E-01	-2.9	41.8	6.0	94.4
2-Piperidinone	16.3	1E-16	16.0	1E-15	3E+00	1.5	30.8	8.6	98.1
(Z)-1,3-Octadiene	-14.6	2E-15	14.7	2E-14	3E-01	-2.0	49.2	8.0	97.9
L-Proline	-14.6	2E-15	14.7	2E-14	6E-01	-0.7	41.5	6.8	96.6
5-Decenoylcarnitine	14.2	4E-15	14.4	4E-14	7E+00	2.8	73.3	6.6	94.7
2-Fluoro-2',3'-dideoxyadenosine	-14.1	5E-15	14.3	4E-14	3E-01	-2.0	44.6	-5.7	97.6
3,8-Dihydroxytetradecanoylcarnitine	14.0	6E-15	14.3	4E-14	2E+02	7.6	74.6	7.8	94.0
N-Acetyl-L-phenylalanine	-13.6	1E-14	13.9	1E-13	4E-01	-1.4	37.5	8.3	96.8
L-alpha-Amino-1H-pyrrole-1-hexanoic acid	13.5	2E-14	13.8	1E-13	7E+01	6.1	56.2	4.7	96.3
Cyclopentanone	-13.4	2E-14	13.7	1E-13	4E-01	-1.2	32.9	9.1	97.6
4-Hydroxy-2,6,6-trimethyl-3-oxo-1,4-cyclohexadiene-1-carboxaldehyde	13.3	2E-14	13.6	2E-13	3E+00	1.7	59.6	4.2	96.9
2-Hydroxy-p-mentha-1,8-dien-6-one	13.3	2E-14	13.6	2E-13	3E+00	1.7	79.9	5.5	96.2
Styrene	-13.2	3E-14	13.5	2E-13	4E-01	-1.3	60.5	7.7	95.0
1-Nitro-7-glutathionyl-8-hydroxy-7,8-dihydronaphthalene	-12.9	5E-14	13.3	3E-13	3E-01	-1.8	66.9	3.4	92.4
Estriol	-12.9	5E-14	13.3	3E-13	3E-01	-1.9	62.6	1.2	94.6
Tryptophyl-Asparagine	-12.9	5E-14	13.3	3E-13	6E-02	-4.0	56.6	-1.2	93.3
N-[(3s)-2-Oxotetrahydrofuran-3-Yl]butanamide	-12.4	2E-13	12.8	9E-13	2E-01	-2.5	31.3	6.2	97.4
(3Z,5E,7E)-Decatrienoylcarnitine	12.4	2E-13	12.8	9E-13	2E+00	1.2	44.5	3.7	88.5
Thioproline	-12.1	3E-13	12.6	1E-12	2E-02	-5.8	25.8	7.0	95.5
3-Hydroxytetradecanoyl carnitine	12.1	3E-13	12.5	2E-12	3E+01	4.8	78.5	8.5	96.0
Terbutaline	-11.8	5E-13	12.3	3E-12	3E-01	-1.6	31.6	6.3	96.4
Rivularine	-11.6	9E-13	12.0	5E-12	5E-02	-4.2	34.3	6.7	96.3
Cyclohexanone	-11.5	1E-12	12.0	5E-12	4E-01	-1.2	41.8	8.0	98.2
2,3-Dimethyl-2-cyclohexen-1-one	-11.4	1E-12	11.9	6E-12	9E-02	-3.4	59.7	6.1	95.4
Tetradeca-9,11-dienedioylcarnitine	11.2	2E-12	11.7	8E-12	9E+00	3.2	35.8	1.8	92.7
Epinephrine glucuronide	-11.2	2E-12	11.7	9E-12	2E-04	-12.3	61.8	1.7	94.8
Prostaglandin F-main urinary metabolite	11.1	2E-12	11.6	1E-11	3E+03	11.5	74.0	1.7	96.0
3-(4-Isopropylphenyl)propanal	10.8	5E-12	11.3	2E-11	4E+00	2.1	47.0	4.7	91.6
3-Epinobilin	10.7	7E-12	11.2	3E-11	4E+02	8.8	72.3	2.2	97.4
butenoylcarnitine	-10.6	7E-12	11.1	3E-11	1E-01	-2.8	23.8	5.9	98.8
Glutamylisoleucine	10.5	9E-12	11.0	4E-11	4E+00	2.1	75.5	1.5	93.8
Adenosine	10.1	2E-11	10.6	9E-11	2E+00	1.0	50.3	7.8	96.5

Supplementary Table 2: Important features identified by two-sided t-tests from the urine of *Slc28a1* mice, including the fragmentation and metabolite identification information and fold-changes ($Slc28a1^{-/-}$ / $Slc28a1^{+/+}$) *FDR: False Discovery Rate

Supplementary Table 3

Accepted Description	t.stat	p.value	minusLOG10(p)	FDR	Fold Change	log2(FC)	Score	Frag. Score	Mass Error (ppm)	Isotope Similarity
L-Tryptophan	5.0	2.0E-05	4.7	5.6E-04	1.2	0.3	47.8	50.3	7.4	97.0
Hypoxanthine	-4.9	3.1E-05	4.5	5.6E-04	0.8	-0.4	45.3	36.9	7.0	97.7
D-Pipecolic acid	-4.9	3.1E-05	4.5	5.6E-04	0.5	-0.9	45.5	40.0	4.9	93.4
Indoleacrylic acid	4.8	3.8E-05	4.4	5.6E-04	1.2	0.3	47.8	50.8	7.7	96.9
4-Hydroxyquinoline	4.5	9.6E-05	4.0	1.0E-03	1.2	0.3	50.6	61.3	4.1	96.6
Arabinosylhypoxanthine	-4.4	1.0E-04	4.0	1.0E-03	0.8	-0.4	46.5	39.8	3.0	96.4
Nicotyrine	4.3	1.5E-04	3.8	1.3E-03	1.2	0.2	53.0	72.6	3.4	96.6
Corticosterone	3.9	4.2E-04	3.4	3.1E-03	1.5	0.6	54.9	82.5	2.5	94.9
3a,4,5,6,7,7a-Hexahydroindene-1,2,3-trione	3.8	5.8E-04	3.2	3.1E-03	2.1	1.0	42.1	21.8	2.5	91.8
1,4-Ipomeadiol	3.8	6.2E-04	3.2	3.1E-03	3.7	1.9	44.4	31.1	4.6	96.5
(E,E)-2,6-Octadienal	3.8	6.8E-04	3.2	3.1E-03	3.6	1.9	45.8	39.9	5.6	95.5
3-Hydroxytetradecanedioic acid	3.8	6.9E-04	3.2	3.1E-03	3.7	1.9	50.2	61.6	6.4	96.4
4-Heptenoic acid	3.8	7.0E-04	3.2	3.1E-03	3.8	1.9	46.4	42.6	5.8	95.8
Methylenecyclohexane	3.7	7.3E-04	3.1	3.1E-03	4.3	2.1	42.8	27.2	6.0	93.7
3-[(2-Mercapto-1-methylpropyl)thio]-2-butanol	-3.6	1.0E-03	3.0	3.9E-03	0.6	-0.6	46.3	46.7	-1.0	85.8
6-Hydroxypentadecanedioic acid	3.5	1.4E-03	2.9	5.0E-03	3.8	1.9	49.7	58.1	2.8	93.9
Hippuric acid	-3.5	1.4E-03	2.8	5.0E-03	0.6	-0.8	43.6	29.0	3.8	93.4
3-Cyclopentylpropionic acid	3.3	2.2E-03	2.7	7.3E-03	3.2	1.7	47.8	41.7	1.3	98.8
2-Methylindole	3.2	2.9E-03	2.5	8.7E-03	1.2	0.2	51.5	64.8	3.9	97.3
xi-7-Hydroxyhexadecanedioic acid	3.2	2.9E-03	2.5	8.7E-03	4.0	2.0	48.9	54.1	2.2	93.2
Pantothenic acid	3.2	3.1E-03	2.5	8.8E-03	1.5	0.6	52.4	70.5	4.1	96.2
6-Hydrazinonicotinic acid	-3.2	3.4E-03	2.5	9.0E-03	0.6	-0.8	49.0	59.8	-9.3	95.5
(3S,5R,6R,7E)-3,5,6-Trihydroxy-7-megastigmen-9-one	3.1	3.8E-03	2.4	9.9E-03	1.7	0.8	52.5	69.5	3.3	96.7
Creatinine	-3.1	4.2E-03	2.4	1.0E-02	0.6	-0.7	40.1	11.2	6.5	96.7
imolamine	3.1	4.6E-03	2.3	1.0E-02	1.7	0.8	45.2	31.5	0.0	94.5
N-Hexadecanoyl-serine	-3.0	4.7E-03	2.3	1.0E-02	0.8	-0.3	46.4	41.2	0.7	91.6
Octyl gallate	3.0	4.8E-03	2.3	1.0E-02	1.4	0.4	45.4	41.2	-6.7	93.2
Sebacic acid	3.0	4.8E-03	2.3	1.0E-02	1.9	0.9	46.5	42.6	2.6	93.1
8-Hydroxy-5,6-octadienoic acid	3.0	5.4E-03	2.3	1.1E-02	1.6	0.7	40.0	10.4	3.8	94.2
Glutamylleucylarginine	3.0	5.8E-03	2.2	1.1E-02	1.9	0.9	47.1	49.3	8.8	96.1
alpha-Campholonic acid	3.0	5.9E-03	2.2	1.1E-02	1.9	0.9	47.1	44.2	3.7	95.9
met-lys-lys	2.9	6.3E-03	2.2	1.1E-02	1.7	0.7	53.3	86.2	-9.5	90.9
N-Eicosapentaenoyl Methionine	2.9	6.4E-03	2.2	1.1E-02	1.9	0.9	45.7	50.8	8.1	86.6
(2R,3S)-3-(6-Amino-9H-purin-9-yl)nonan-2-ol	2.9	6.6E-03	2.2	1.1E-02	1.6	0.7	46.1	43.0	-3.3	91.6
20-Trihydroxy-leukotriene-B4	2.9	7.3E-03	2.1	1.2E-02	1.6	0.7	44.5	31.8	0.8	91.6
Butanedioic acid, octenyl-	2.8	7.9E-03	2.1	1.3E-02	1.6	0.6	46.8	43.8	5.7	96.7
Dynorphin B (10-13)	2.8	8.1E-03	2.1	1.3E-02	1.5	0.6	43.5	33.5	-3.3	88.1
3-Hydroxydodecanedioic acid	2.8	8.5E-03	2.1	1.3E-02	1.5	0.6	48.2	51.5	6.1	96.5
1-Benzazepine	2.8	8.6E-03	2.1	1.3E-02	1.2	0.2	51.8	66.5	1.9	94.8
9,13-Dihydroxy-4-megastigmen-3-one 9-glucoside	2.8	8.7E-03	2.1	1.3E-02	1.6	0.7	53.0	75.0	3.1	93.5
asn-gly-lys-gly	2.6	1.3E-02	1.9	1.8E-02	1.6	0.7	47.5	54.2	7.9	92.3
L-Tyrosine	2.6	1.4E-02	1.9	2.0E-02	1.3	0.3	53.1	73.5	4.8	97.3
Creatine	-2.6	1.5E-02	1.8	2.0E-02	0.7	-0.5	44.3	33.7	8.9	97.7
Isocrotonic acid	2.6	1.5E-02	1.8	2.0E-02	1.5	0.6	40.5	13.8	7.8	97.2
13-Docosenamide	2.6	1.6E-02	1.8	2.0E-02	1.6	0.6	51.4	63.9	0.9	94.5
2,5-Diamino-4,5-diketopyrimidine	2.5	1.6E-02	1.8	2.1E-02	1.2	0.3	47.9	42.4	-1.5	99.1
8-Aminooctanoic acid	-2.5	1.8E-02	1.7	2.2E-02	0.6	-0.7	48.1	50.0	6.2	97.5
N-Eicosapentaenoyl Cysteine	2.5	1.8E-02	1.7	2.2E-02	1.5	0.6	42.5	35.6	7.0	84.6
(3R,7R)-1,3,7-Octanetriol	2.4	2.1E-02	1.7	2.6E-02	1.2	0.2	42.3	20.1	5.3	97.6
2-Ethyl-3,4-dihydroxyfuran	2.3	2.6E-02	1.6	3.0E-02	1.3	0.4	43.9	37.5	-5.2	88.0
LysoPC(18:3(6Z,9Z,12Z)/0:0)	-2.3	2.9E-02	1.5	3.3E-02	0.8	-0.2	52.6	79.9	-2.6	86.3
Propionylcarnitine	-2.1	4.7E-02	1.3	5.3E-02	0.8	-0.4	46.8	43.1	4.8	96.6
Hexanoylcarnitine	-1.9	6.5E-02	1.2	7.0E-02	0.7	-0.5	45.5	35.5	3.5	95.9
Oxidized glutathione	-1.7	1.1E-01	1.0	1.1E-01	0.7	-0.6	44.9	40.3	3.0	87.6
Pentadeca-5,7,9-trienedioylcarnitine	-1.4	1.6E-01	0.8	1.7E-01	0.8	-0.3	42.1	26.6	9.3	94.0
Adenosine monophosphate	-0.7	5.1E-01	0.3	5.2E-01	0.7	-0.5	46.4	39.7	1.6	94.4
Adenosine	-0.5	6.0E-01	0.2	6.0E-01	0.9	-0.2	49.3	54.6	4.2	96.6

Supplementary Table 3. Important features identified by two-sided t-tests from the plasma of *Slc28a1* mice, including the fragmentation and metabolite identification information and fold-changes (*Slc28a1*$^{-/-}$ / *Slc28a1*$^{+/+}$) *FDR: False Discovery Rate

Supplementary Table 4

Urine

Table 3: Meta-Analysis of Mummichog and GSEA Results

	Total_Size	Hits	Sig_Hits	Mummichog_Pvals	GSEA_Pvals	Combined_Pvals
Arginine and proline metabolism	37.00	18.00	17.00	0.13	0.03	0.03
Citrate cycle (TCA cycle)	16.00	6.00	6.00	0.30	0.02	0.03
Valine, leucine and isoleucine biosynthesis	8.00	8.00	8.00	0.20	0.06	0.07
Pyrimidine metabolism	39.00	12.00	11.00	0.33	0.04	0.07
Aminoacyl-tRNA biosynthesis	22.00	15.00	14.00	0.21	0.07	0.08
Steroid hormone biosynthesis	77.00	54.00	49.00	0.05	0.33	0.08
Metabolism of xenobiotics by cytochrome P450	64.00	18.00	10.00	1.00	0.02	0.09
Purine metabolism	66.00	21.00	13.00	0.99	0.02	0.11
Glutathione metabolism	19.00	6.00	5.00	0.71	0.07	0.20
Terpenoid backbone biosynthesis	15.00	1.00	1.00	0.82	0.09	0.26
D-Glutamine and D-glutamate metabolism	6.00	6.00	6.00	0.30	0.26	0.28
Retinol metabolism	15.00	8.00	5.00	0.96	0.10	0.33
Pantothenate and CoA biosynthesis	17.00	7.00	7.00	0.25	0.41	0.33
Alanine, aspartate and glutamate metabolism	28.00	9.00	9.00	0.16	0.67	0.35
Porphyrin and chlorophyll metabolism	27.00	8.00	7.00	0.56	0.21	0.37
Arachidonic acid metabolism	35.00	34.00	17.00	1.00	0.13	0.39
Tyrosine metabolism	42.00	29.00	25.00	0.38	0.35	0.40
Phenylalanine metabolism	12.00	9.00	9.00	0.16	0.88	0.42
Valine, leucine and isoleucine degradation	35.00	13.00	12.00	0.29	0.58	0.47
Butanoate metabolism	15.00	8.00	6.00	0.84	0.22	0.50
Glycine, serine and threonine metabolism	31.00	11.00	9.00	0.69	0.28	0.51
Lysine degradation	19.00	5.00	5.00	0.37	0.52	0.51
Phenylalanine, tyrosine and tryptophan biosynthesis	4.00	3.00	3.00	0.55	0.40	0.55
Ascorbate and aldarate metabolism	10.00	1.00	1.00	0.82	0.30	0.60
Thiamine metabolism	5.00	1.00	1.00	0.82	0.32	0.62
Vitamin B6 metabolism	9.00	4.00	4.00	0.45	0.62	0.64
Fatty acid biosynthesis	10.00	1.00	1.00	0.82	0.35	0.64
Nitrogen metabolism	6.00	2.00	2.00	0.67	0.43	0.65
One carbon pool by folate	9.00	6.00	5.00	0.71	0.43	0.66
Histidine metabolism	16.00	6.00	5.00	0.71	0.43	0.67
Arginine biosynthesis	14.00	4.00	4.00	0.45	0.74	0.70
Ubiquinone and other terpenoid-quinone biosynthesis	9.00	4.00	3.00	0.85	0.40	0.70
Folate biosynthesis	24.00	10.00	6.00	0.98	0.35	0.71
Tryptophan metabolism	41.00	20.00	16.00	0.72	0.49	0.72
Glycolysis or Gluconeogenesis	23.00	1.00	1.00	0.82	0.46	0.74
Propanoate metabolism	19.00	7.00	5.00	0.89	0.43	0.75
alpha-Linolenic acid metabolism	12.00	2.00	2.00	0.67	0.61	0.78
Nicotinate and nicotinamide metabolism	15.00	4.00	2.00	0.98	0.46	0.81
Glycerophospholipid metabolism	13.00	2.00	2.00	0.67	0.67	0.81
Biosynthesis of unsaturated fatty acids	34.00	6.00	5.00	0.71	0.65	0.82
Cysteine and methionine metabolism	33.00	7.00	6.00	0.63	0.74	0.82
Drug metabolism - cytochrome P450	21.00	10.00	8.00	0.74	0.65	0.84
Glyoxylate and dicarboxylate metabolism	31.00	7.00	6.00	0.63	0.77	0.84
Taurine and hypotaurine metabolism	8.00	3.00	3.00	0.55	0.90	0.84
Synthesis and degradation of ketone bodies	5.00	2.00	1.00	0.97	0.53	0.85
Biotin metabolism	4.00	1.00	1.00	0.82	0.65	0.87
Phosphonate and phosphinate metabolism	4.00	1.00	1.00	0.82	0.65	0.87
N-Glycan biosynthesis	38.00	2.00	1.00	0.97	0.61	0.90
Primary bile acid biosynthesis	46.00	7.00	6.00	0.63	0.96	0.91
Riboflavin metabolism	4.00	1.00	1.00	0.82	0.95	0.97
Selenocompound metabolism	16.00	1.00	1.00	0.82	0.96	0.97
D-Arginine and D-ornithine metabolism	4.00	1.00	1.00	0.82	0.96	0.98
Pyruvate metabolism	19.00	4.00	2.00	0.98	0.81	0.98
beta-Alanine metabolism	21.00	5.00	3.00	0.96	0.96	1.00
Amino sugar and nucleotide sugar metabolism	35.00	4.00	2.00	0.98	0.94	1.00

Supplementary Table 4. Mummichog and GSEA pathway analysis results table from *Slc28a1*$^{-/-}$ urine**.** Table displays ranked pathways that are enriched in *Slc28a1*$^{-/-}$mice. The table displays matched pathways, the total number of hits per pathway, the raw p-values (*p < 0.05 by one-tailed hypergeometric test) for each algorithm, and the combined p-values (*p < 0.05 by two-tailed Welch’s t test).

Supplementary Table 5

Plasma

Table 3: Meta-Analysis of Mummichog and GSEA Results

	Total_Size	Hits	Sig_Hits	Mummichog_Pvals	GSEA_Pvals	Combined_Pvals
Steroid hormone biosynthesis	77.00	25.00	5.00	0.39	0.01	0.04
Tryptophan metabolism	41.00	5.00	3.00	0.03	0.22	0.04
Arginine and proline metabolism	37.00	12.00	2.00	0.62	0.03	0.10
Tyrosine metabolism	42.00	16.00	3.00	0.51	0.04	0.11
Riboflavin metabolism	4.00	1.00	1.00	0.16	0.23	0.16
Purine metabolism	66.00	14.00	2.00	0.71	0.06	0.18
Caffeine metabolism	12.00	5.00	1.00	0.60	0.14	0.29
Glycine, serine and threonine metabolism	31.00	6.00	1.00	0.66	0.18	0.38
Glycerophospholipid metabolism	13.00	2.00	1.00	0.30	0.47	0.42
Primary bile acid biosynthesis	46.00	3.00	1.00	0.42	0.57	0.58
Phenylalanine metabolism	12.00	6.00	1.00	0.66	0.46	0.67
Lysine degradation	19.00	4.00	1.00	0.52	0.67	0.71
Aminoacyl-tRNA biosynthesis	22.00	14.00	1.00	0.93	0.37	0.71
Linoleic acid metabolism	4.00	4.00	1.00	0.52	0.81	0.78
Porphyrin and chlorophyll metabolism	27.00	4.00	1.00	0.52	0.81	0.78
Pantothenate and CoA biosynthesis	17.00	7.00	1.00	0.72	0.58	0.78
Arachidonic acid metabolism	35.00	14.00	1.00	0.93	0.70	0.93

Supplementary Table 5. Mummichog and GSEA pathway analysis results table from *Slc28a1*$^{-/-}$ plasma**.** Table displays ranked pathways that are enriched in *Slc28a1*$^{-/-}$mice. The table displays matched pathways, the total number of hits per pathway, the raw p-values (*p < 0.05 by one-tailed hypergeometric test) for each algorithm, and the combined p-values (*p < 0.05 by two-tailed Welch's t test).
