# Supplementary material for: Increased renal elimination of endogenous and synthetic pyrimidine nucleosides in concentrative nucleoside transporter 1 deficient mice
Source: Nat Commun. 2023 Jun 1;14:3175. doi: 10.1038/s41467-023-38789-8 (PMC10235067; doi:10.1038/s41467-023-38789-8)
Supplement: Supplementary file 2 — Description of Additional Supplementary Files [file 41467_2023_38789_MOESM2_ESM.pdf]

Description of Additional Supplementary Files

Supplementary Data 1. Untargeted metabolomic profiling of the *Slc28a1*$^{-/-}$ mouse urine samples: Top altered metabolites

Supplementary Data 2. Untargeted metabolomic profiling of the *Slc28a1*$^{-/-}$ mouse plasma samples: Top altered metabolites
