# Supplementary material for: Increased renal elimination of endogenous and synthetic pyrimidine nucleosides in concentrative nucleoside transporter 1 deficient mice
Source: Nat Commun. 2023 Jun 1;14:3175. doi: 10.1038/s41467-023-38789-8 (PMC10235067; doi:10.1038/s41467-023-38789-8)
Supplement: Supplementary file 5 — Reporting Summary [file 41467_2023_38789_MOESM5_ESM.pdf]

nature portfolio

Corresponding author(s): Rajgopal Govindarajan

Last updated by author(s): Apr 26, 2023

Reporting Summary

Nature Portfolio wishes to improve the reproducibility of the work that we publish. This form provides structure for consistency and transparency in reporting. For further information on Nature Portfolio policies, see our Editorial Policies and the Editorial Policy Checklist.

Statistics

For all statistical analyses, confirm that the following items are present in the figure legend, table legend, main text, or Methods section.

n/a	Confirmed	
☐	☒	The exact sample size (*n*) for each experimental group/condition, given as a discrete number and unit of measurement
☐	☒	A statement on whether measurements were taken from distinct samples or whether the same sample was measured repeatedly
☐	☒	The statistical test(s) used AND whether they are one- or two-sided<br>*Only common tests should be described solely by name; describe more complex techniques in the Methods section.*
☒	☐	A description of all covariates tested
☒	☐	A description of any assumptions or corrections, such as tests of normality and adjustment for multiple comparisons
☐	☒	A full description of the statistical parameters including central tendency (e.g. means) or other basic estimates (e.g. regression coefficient) AND variation (e.g. standard deviation) or associated estimates of uncertainty (e.g. confidence intervals)
☐	☒	For null hypothesis testing, the test statistic (e.g. *F*, *t*, *r*) with confidence intervals, effect sizes, degrees of freedom and *P* value noted<br>*Give P values as exact values whenever suitable.*
☒	☐	For Bayesian analysis, information on the choice of priors and Markov chain Monte Carlo settings
☒	☐	For hierarchical and complex designs, identification of the appropriate level for tests and full reporting of outcomes
☐	☒	Estimates of effect sizes (e.g. Cohen's *d*, Pearson's *r*), indicating how they were calculated

Our web collection on statistics for biologists contains articles on many of the points above.

Software and code

Policy information about availability of computer code

Data collection	No software was used for data collection
Data analysis	MassLynx 4.1 Software AutoTune Wizard, Metaboanalyst 4.0, Progenesis QI 3.0 for mass spectrometry data statistical analysis, WinNonlin 6.2 software for pharmacokinetic studies, Living Image software 4.8.0 for bioluminescent imaging, Graphpad Prism 9 for all other statistical analyses, and Image J software 1.5.1 for Western blotting analysis, were used in the study.

For manuscripts utilizing custom algorithms or software that are central to the research but not yet described in published literature, software must be made available to editors and reviewers. We strongly encourage code deposition in a community repository (e.g. GitHub). See the Nature Portfolio guidelines for submitting code & software for further information.

Data

Policy information about availability of data

All manuscripts must include a data availability statement. This statement should provide the following information, where applicable:

- Accession codes, unique identifiers, or web links for publicly available datasets
- A description of any restrictions on data availability
- For clinical datasets or third party data, please ensure that the statement adheres to our policy

Metabolomics data have been deposited in the EMBL-EBI MetaboLights database with the study identifier MTBLS1768.

Human research participants

Policy information about studies involving human research participants and Sex and Gender in Research.

Reporting on sex and gender	The study did not involve human research participants.
Population characteristics	n/a
Recruitment	n/a
Ethics oversight	n/a

Note that full information on the approval of the study protocol must also be provided in the manuscript.

Field-specific reporting

Please select the one below that is the best fit for your research. If you are not sure, read the appropriate sections before making your selection.

- [x] Life sciences
- [ ] Behavioural & social sciences
- [ ] Ecological, evolutionary & environmental sciences

For a reference copy of the document with all sections, see nature.com/documents/nr-reporting-summary-flat.pdf

Life sciences study design

All studies must disclose on these points even when the disclosure is negative.

Sample size	For all in vitro experiments, a sample size of n=3-12 was used based on the effect size and overlap between distributions. A power of 0.8 was set as minimal to decide the sample size for each experiment. With alpha=0.05 and power=0.9 and allowing for unexpected mortalities of ~5%/group, ≥5 mice per group were needed for in vivo studies. For in vivo studies, a sample size of n=6 independent mice per group was used to include inherent variabilities in survival times among experimental mice based on previous pilot studies that yielded high power (>0.8). The sample size for the targeted metabolomics experiments was calculated based on the power analysis module in Metaboanalyst software, which uses algorithms described by van Iterson et al. 2013. The desired power of 0.8 was achieved for the metabolomics data validating the sample size of n=9 independent mice per group.
Data exclusions	No data were excluded from the analysis.
Replication	All experimental findings were reliably reproduced, and attempts at replication of experimental findings were successful. Both in vitro and in vivo experiments were replicated at least two to three times to confirm experimental trends prior to publication.
Randomization	For all studies using mice and mouse tissues, animals of either sex and similar age were randomly arranged into groups. This allocation method allowed us to generate homogeneous blocks for a randomized block design. For immunohistochemistry and clinical/anatomic pathology, the field of view was randomly selected for analysis. For immunoblotting, RT-qPCR, transport studies, and metabolite profiling, samples were randomized and analyzed with standard approaches.
Blinding	Endpoint measures and outcomes for all animal experiments were objective (survival times) that did not necessitate blinding in animal experiments. For in vitro studies, the experimental groups and data collection were blind. However, the data were then analyzed without blinding since we need to know each experimental condition to analyze samples as groups.

Reporting for specific materials, systems and methods

We require information from authors about some types of materials, experimental systems and methods used in many studies. Here, indicate whether each material, system or method listed is relevant to your study. If you are not sure if a list item applies to your research, read the appropriate section before selecting a response.

Materials & experimental systems

n/a	Involved in the study	
☐	☒	Antibodies
☐	☒	Eukaryotic cell lines
☒	☐	Palaeontology and archaeology
☐	☒	Animals and other organisms
☒	☐	Clinical data
☒	☐	Dual use research of concern

Methods

n/a	Involved in the study	
☒	☐	ChIP-seq
☒	☐	Flow cytometry
☒	☐	MRI-based neuroimaging

Antibodies

Antibodies used	For Western blotting analysis, the following antibodies were used: Primary Antibodies - mCNT1 (Alomone Labs; ANT-061, 1:500) reacts with human, rat and mouse variants and was validated by the manufacturer for use in Western blotting using the mouse tissues. GAPDH (CST 97166, 1:5000) reacts with human, rat, mouse and monkey variants and was validated by the manufacturer for use in Western blotting using various cell lines. Secondary Antibodies- Rabbit (Bethyl A120-201P,1:5,000) and mouse (Bethyl A90-116P, 1:5,000) secondary antibodies were used. Note: The source of these antibodies and catalog numbers are provided in parenthesis.
Validation	All antibodies have been validated by the manufacturer using a series of cell lines and mouse tissues. mCNT1-Western blotting analysis of rat kidney, rat liver and mouse kidney lysates (Cell Signaling Technology) GAPDH-Western blotting analysis in HeLa, NIH-3T3, C6, COS-7 cell lines (Alomone Labs).

Eukaryotic cell lines

Policy information about cell lines and Sex and Gender in Research

Cell line source(s)	Pancreatic tumor cells derived from a PDX-1-CRE, LSL-KRasG12D, LSL-Trp53-/- (KPC) genetically engineered mouse model (GEMM) transfected with enhanced firefly luciferase (KPC-LUC) were provided by Dr. Cruz-Monserrate at the Ohio State University. HEK293T Cell Line was procured from ATCC (#CRL 1573). These cell lines were propagated, expanded, and frozen immediately upon receipt. The cells revived from the frozen stock were used within 10-20 passages, not exceeding a period of 2-3 months.
Authentication	The cells were subjected to morphological, cytogenetic, and DNA profile analyses for the characterization of cell lines.
Mycoplasma contamination	All cell lines tested negative for mycoplasma contamination.
Commonly misidentified lines (See ICLAC register)	No commonly misidentified cell lines were used in the study.

Animals and other research organisms

Policy information about studies involving animals; ARRIVE guidelines recommended for reporting animal research, and Sex and Gender in Research

Laboratory animals	Mouse C57BL/6NTac zygotes (Taconic; Rensselaer, NY) were used for CRISPR-Cas9-mediated generation of Slc28a1-/- mice which were backcrossed to C57BL/6NTac WT animals for three generations before breeding the homozygous animals used for subsequent experiments. Genotyping of 3-week old animals was performed followed by subsequent experimental procedures at 8-20 weeks of age. Mouse plasma and urine were used for metabolomics and biochemical studies. Mouse tissue lysates were used for Western blotting and formalin-fixed paraffin-embedded tissue sections were used for immunohistochemistry experiments. Mice were housed in a conventional animal facility with an ambient temperature of 20-22°C, humidity 40%-60%, with a 12-h light/dark cycle and were given free access to standard rodent chow and water.
Wild animals	The study did not involve wild animals.
Reporting on sex	Sex-based analysis was not performed.
Field-collected samples	The study did not involve samples collected from the field.
Ethics oversight	All animal studies were performed in accordance with Animal Care and Use Programs under protocols approved by the Institutional Animal Care and Use Committee at OSU. We have complied with the relevant ethical considerations for animal research overseen by this committee.

Note that full information on the approval of the study protocol must also be provided in the manuscript.
